# Microwave-assisted synthesis and *in vitro* and *in silico* studies of pyrano[3,2-*c*]quinoline-3-carboxylates as dual acting anti-cancer and anti-microbial agents and potential topoisomerase II and DNA-gyrase inhibitors†

**DOI:** 10.1039/d4ra06201a

**Published:** 2025-01-21

**Authors:** Ashraf A. Aly, Hisham A. Abd El-Naby, Essam Kh. Ahmed, Sageda A. Gedamy, Kari Rissanen, Martin Nieger, Alan B. Brown, Michael G. Shehat, Marwa M. Shaaban, Amal Atta

**Affiliations:** a Chemistry Department, Faculty of Science, Minia University 61519 El-Minia Egypt ashrafaly63@yahoo.com ashraf.shehata@mu.edu.eg hisham_minia@mu.edu.eg essam.mohd@mu.edu.eg sagedaali332@yahoo.com; b Department of Chemistry, University of Jyväskylä P. O. Box 35 FIN-40014 Jyväskylä Finland kari.t.rissanen@jyu.fi; c Department of Chemistry, University of Helsinki P. O. Box 55, A. I. Virtasen aukio I 00014 Helsinki Finland martin.nieger@helsinki.fi; d Department of Chemistry and Chemical Engineering, Florida Institute of Technology Melbourne FL 32901 USA abrown@fit.edu; e Department of Microbiology, Faculty of Pharmacy, Alexandria University Alexandria 21521 Egypt michael.shehat@alexu.edu.eg; f Department of Pharmaceutical Chemistry, Faculty of Pharmacy, Alexandria University Alexandria 21521 Egypt maraw.mamdouh@alexu.edu.eg amal.atta@alexu.edu.eg missalex_pharma@yahoo.com

## Abstract

A microwave-assisted method was utilized to synthesize novel pyranoquinolone derivatives as dual acting topoisomerase II/DNA gyrase inhibitors with apoptosis induction ability for halting lung cancer and staphylococcal infection. Herein, the designed rationale was directed toward mimicking the structural features of both topoisomerase II and DNA gyrase inhibitors as well as endowing them with apoptosis induction potential. The absolute configuration of the series was assigned using X-ray diffraction analysis. Cytotoxic activity against NSCLC A549 cells showed that ethyl 2-amino-9-bromo-4-(furan-2-yl)-5-oxo-5,6-dihydro-4*H*-pyrano[3,2-*c*]quinoline-3-carboxylate (IC_50_ ≈ 35 μM) was the most potent derivative in comparison to the positive control Levofloxacin and was selected for further investigation to assess its selectivity (SI = 1.23). Furthermore, *in vitro* antibacterial screening revealed the potential activity of this bromo derivative against *Staphylococcus aureus*. Mechanistic studies showed that the aforementioned compound exhibited promising inhibitory activity against topoisomerase II (IC_50_ = 45.19 μM) and DNA gyrase (IC_50_ = 40.76 μM) compared to reference standards. In addition, the previous compound induced a A549 cell apoptosis by 38.49-fold and it also increased the total apoptosis by 20.4% compared to a 0.53% increase in the control. Docking simulations postulated its interactions and suggested well fitting into its molecular targets.

## Abstract 2

A microwave-assisted method was utilized to synthesize novel pyranoquinolone derivatives as dual acting topoisomerase II/DNA gyrase inhibitors with apoptosis induction ability for halting lung cancer and *staphylococcal* infection.

## Graphical abstract

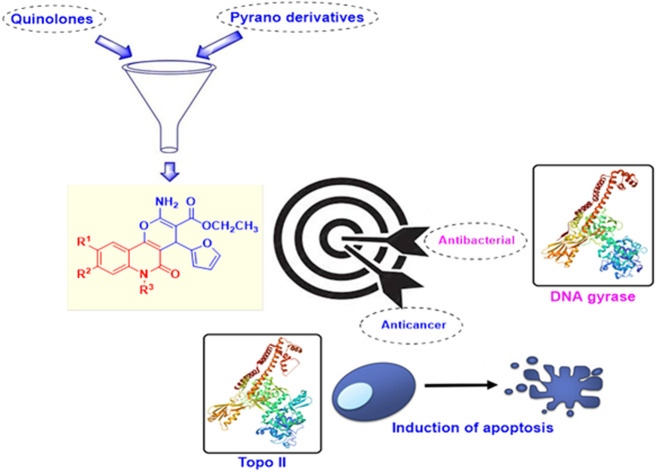

## 1. Introduction

Cancer is a fatal disease characterized by uncontrolled cell proliferation that invades surrounding tissue and is associated with a high mortality rate.^1^ The WHO's global cancer report predicts that the percentage of deaths from cancer will double in the next several years. As a result, many academics are now considered the development of novel effective anticancer medications, it has been considered to be an urgent requirement.^2^ Thus, research on heterocyclic cores possessing anticancer potential has attracted great interest worldwide.^3^

**Fig. 1:**
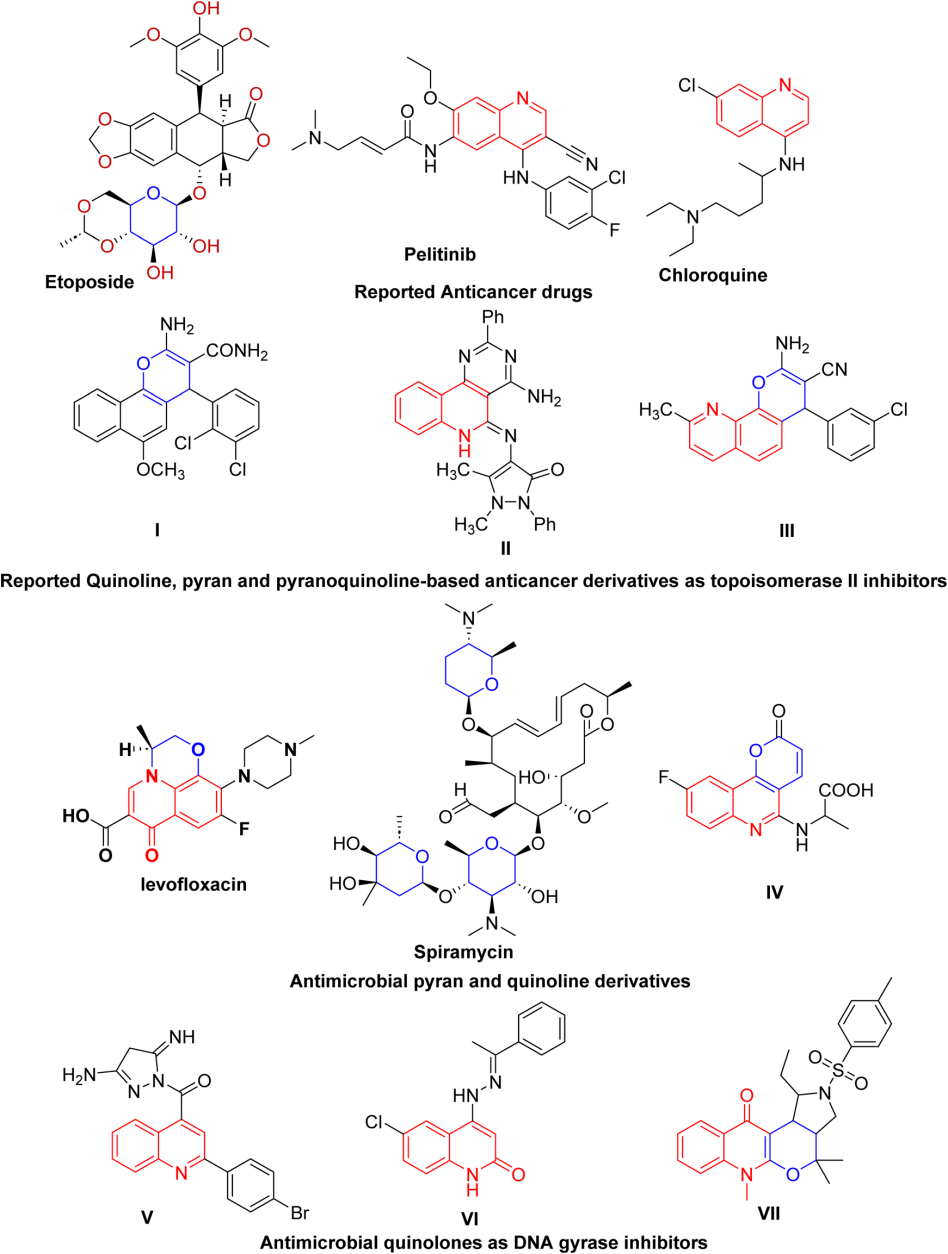
Reported quinoline-, pyran- and pyranoquinoline-based anticancer and antibacterial derivatives as potential inhibitors of topoisomerase II and DNA gyrase.

Globally, lung cancer is the leading cause of cancer-related mortality.^4^ Adjuvant chemotherapy and antineoplastic agents are conventional treatments for advanced non-small cell lung cancer (NSCLC).^5^ Since topoisomerase enzymes are crucial for DNA metabolism, finding enzyme inhibitors is a key goal in the hunt for novel anticancer medications.^6^ They act by inhibiting topoisomerases from relegating DNA strands after cleavage, leading to DNA damage. The majority of anticancer agents are mainly directed toward DNA topoisomerase inhibition.^7^ The development of novel anticancer medications that specifically target topoisomerase II (Topo II) is a source of interest for medicinal chemists to overcome resistance and improve chemotherapeutic outcomes. For instance, etoposide is a significant chemotherapeutic drug inhibiting Topo II, which has been used to treat a variety of human malignancies for over 20 years and is still among the most often prescribed anticancer medications worldwide.^8^ Moreover, quinoline and pyran derivatives exhibit a variety of pharmacological activities, making them important pharmaceutically active heterocyclic compounds.^9–11^ They are important cores for creating new classes of structural entities used in cancer treatment (Fig. 1).^9,12^ The quinoline framework is essential for the development of anticancer drugs *via* a variety of mechanisms, including angiogenesis inhibition, apoptosis induction, growth inhibition by cell cycle arrest, and cell-migration disruption.^13^ In addition, drugs composed of quinolone and pyran rings targeting topoisomerase are currently widely used as frontline medications for the treatment of cancer (Fig. 1).^14^

Patients with cancer are at an increased risk of bacterial and antibiotic-resistant infections compared with healthy individuals.^15^ Particularly, lung cancer patients undergoing chemotherapeutic and surgical treatment are more likely to suffer from pulmonary complications caused mainly by bacterial infection. Regarding the bacterial spectrum of lung cancer patients, *Staphylococcus aureus* is one of the most frequently isolated organisms.^16^ Moreover, growing evidence indicates that staphylococcal infection is closely linked to the incidence and development of several cancer types, signifying that both inflammation and immunity play a certain role in the development of cancer; thus, there is an association between *Staphylococcus* and carcinogenesis.^15,17^ Consequently, bacterial infection may affect the growth of lung cancer by activating inflammatory signaling. *Staphylococcus aureus* lipoteichoic acid (LTA) induced a prominent increase in the cellular growth and proliferation of the NSCLC cell lines A549.^18^ Besides, *Staphylococcus aureus* infection is involved in the regulation of cancer cell metastasis.^19^

Molecular approaches incorporating pyran and quinoline moieties (Fig. 1) have revealed remarkable antimicrobial activity against Gram-negative and Gram-positive bacteria as well as against fungal pathogens.^20–23^ Particularly, quinolones are broad-spectrum antibacterial agents targeting DNA gyrase, a type II topoisomerase. DNA gyrase plays a vital role in transcription and bacterial DNA replication, making it an essential therapeutic target in antibacterial drug discovery. However, adverse effects and emerging drug resistance render the currently available quinolones less effective.^24^ Several quinoline entities (Fig. 1) with diverse scaffolds have been identified as DNA gyrase inhibitors, which could serve as good leads for the development of novel antibacterial agents.^25,26^ Additionally, various synthetic techniques have been utilized to develop new quinolones or to modify the quinolone scaffold with the aim of reducing toxicity and overcoming resistance.^27,28^ In addition, spiramycin, a pyran derivative, is a macrolide antibiotic with activity primarily against *Staphylococcus aureus*.^29^

**Fig. 2:**
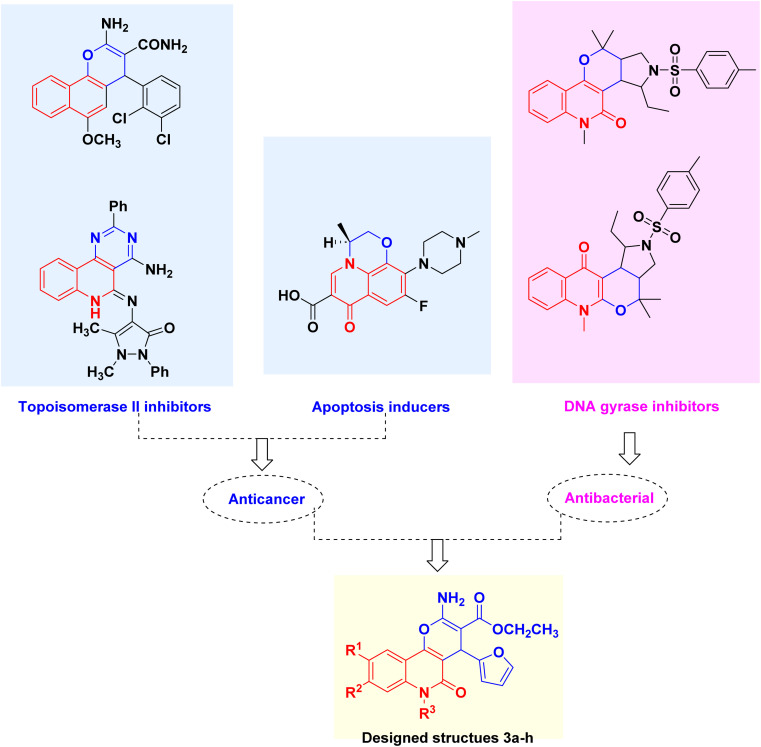
Lead Topo II inhibitors, apoptosis inducers, DNA gyrase inhibitors and design rationale of the multitarget pyranoquinolones 3a–h.

As a follow-up to our work on the synthesis of biologically active-fused quinolones, and in light of the aforementioned activity of pyranes and quinolines, this study aims to combine the pyrane and quinoline scaffolds into a single molecule in addition to modifying the nature of the linked quinoline ring by diversifying the substitution, allowing us to assess the SAR among the investigated series in the quest of promising compounds regarding activity (Fig. 2). Herein, we report a novel series of pyrano[3,2-*c*]quinoline-3-carboxylates combating the staphylococcal infection-lung cancer interplay by inhibiting both DNA gyrase and topoisomerase II with apoptosis induction ability.

## 2. Results and discussion

### 2.1. Chemistry

**Scheme 1:**
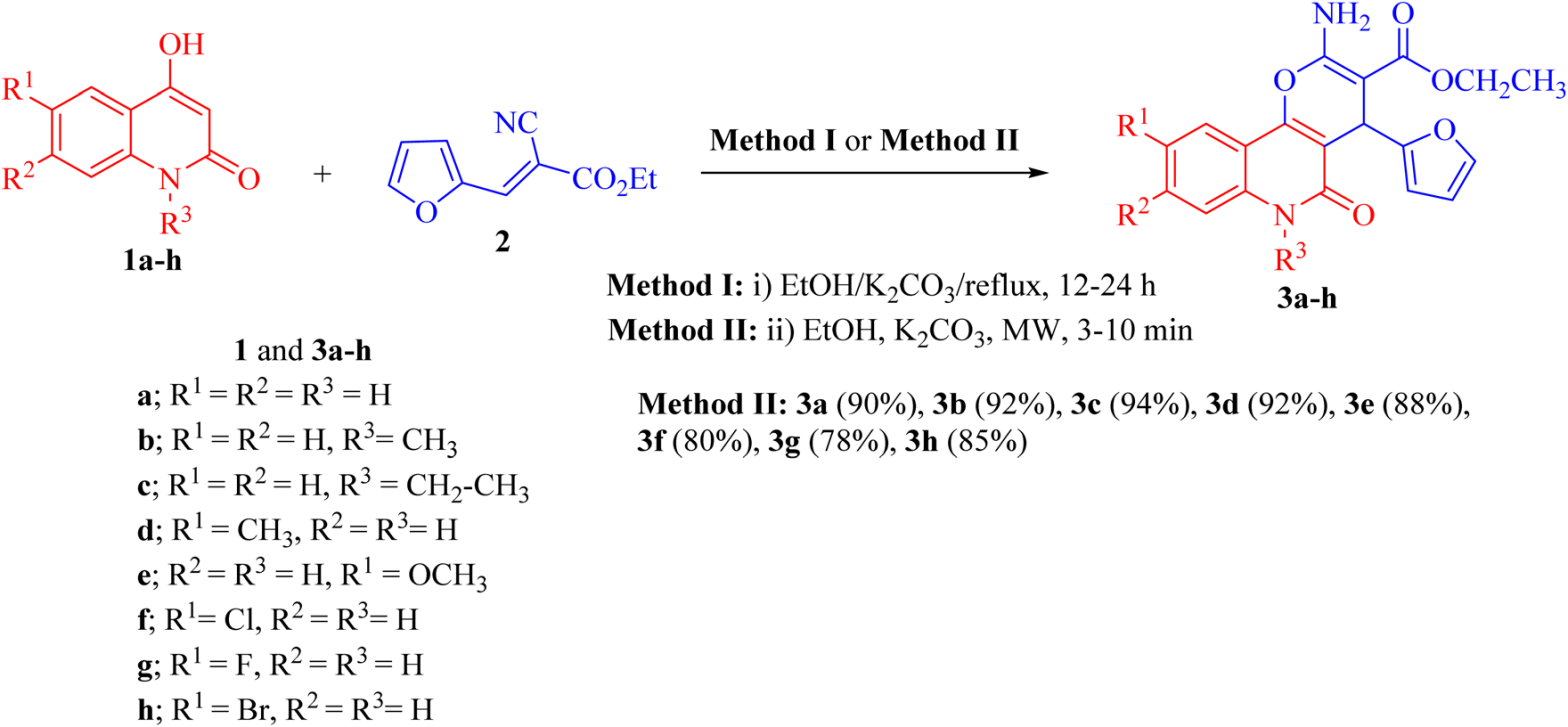
Synthesis of ethyl 2-amino-4-(furan-2-yl)-5-oxo-pyrano[3,2-*c*]quinoline-3- carboxylates 3a–h.

Pyrano[3,2-*c*]quinoline-3-carboxylate derivatives 3a–h were obtained *via* the reaction of 4-hydroxy-2-oxo-1,2-dihydroquinoline derivatives 1a–h with ethyl (*E*)-2-cyano-3-(furan-2-yl)acrylate (2) (Scheme 1). The reaction was performed under different conditions, such as ethanol/K_2_CO_3_ at reflux (Method I) and ethanol/K_2_CO_3_/microwave (Method II) (Scheme 1).

Interestingly, when the reaction was carried out by Method II, the yields of products 3a–j were found to be excellent for some derivatives (75–94%) and also took a shorter time (Table 1). Method II showed that the reaction between 1a–h and 2 was completed faster and gave excellent yields (80–94%) compared with the conventional method (Method I).

**Table 1:** Time and yield of 2-amino-4-(furan-2-yl)-5-oxo-pyrano[3,2-*c*]quinoline-3-carboxylates 3a–h using Methods I and II

Compounds	Time (min or h)		Yield (%)	
	Method I (h)	Method II (min)	Method I	Method II
3a	12	3	75	90
3b	14	4	65	92
3c	15	4	77	94
3d	18	7	80	92
3e	20	6	78	88
3f	26	8	64	80
3g	20	9	65	78
3h	24	10	82	85

**Fig. 3:**
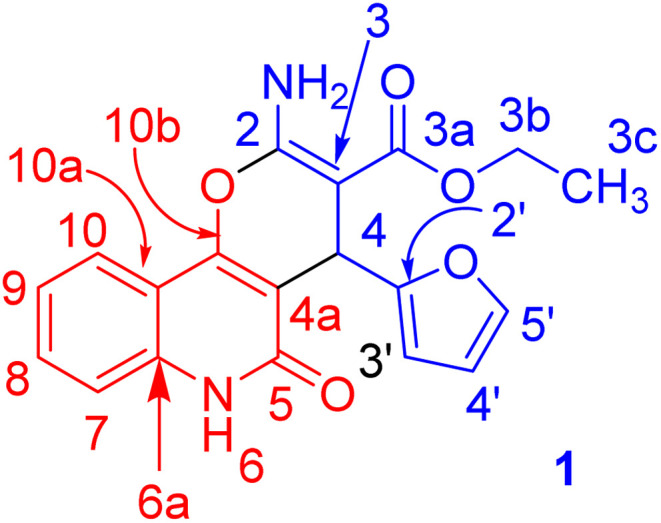
Distinctive carbons of compound 3a.

Under microwave irradiation, compounds 3a, 3b and 3c were formed completely in a short time (>5 min) and afforded yields of 90%, 92% and 94%, respectively. The best yield was obtained in the case of 3c (94%), and the reaction was completed in 4 min (Table 1). The ^1^H NMR spectrum of 3a showed the ethyl protons as a triplet for CH_3_ at *δ*_H_ = 1.21 (*J* = 7.1 Hz) and the CH_2_-ester at *δ*_H_ = 4.11 (**A**BX_3_, *J*_AB_ = 14.2, *J*_AX_ = 7.1 Hz; 1H) and *δ*_H_ = 4.08 ppm (A**B**X_3_, *J*_AB_ = 14.2, *J*_BX_ = 7.1 Hz; 1H). The NH_2_ protons resonated as a broad singlet at *δ*_H_ = 7.77 ppm, while the H-pyran appeared as a singlet at *δ*_H_ = 5.03 ppm. The three protons of furan appeared as a doublet at *δ*_H_ = 6.28 (*J* = 1.8 Hz), a double-doublet at *δ*_H_ = 6.07 (*J* = 2.0, 1.0 Hz) and a doublet at *δ*_H_ = 7.37 ppm (*J* = 0.8 Hz) for H-3′, H-4′ and H-5′, respectively. The ^13^C NMR spectrum confirmed the ^1^H NMR spectral data. As ^13^C NMR spectrum revealed the ethyl-ester carbon signals at *δ*_C_ = 14.3 (CH_3_) and 58.8 (CH_2_), while the CH-pyran appeared as a singlet at *δ*_C_ = 28.2 ppm. The two carbonyl carbon signals (C-3a and C-5) for the ester and quinolyl groups resonated at *δ*_C_ = 167.7 and 156.4 ppm, respectively. The carbon signal of pyran-2-C (C-2) in the ^13^C NMR spectrum appeared at *δ*_C_ = 160.6 ppm. Distinctive carbons are shown in Fig. 3. The NMR spectral data for compound 3a are shown in Table 2.

NMR spectral data of compound 3a

**Table:** 

^1^H NMR (DMSO-*d*_6_)	^1^H–^1^H COSY	Assignment
7.96 (d, *J* = 8.0 Hz; 1H)	*7.58*, 7.30	H-10
7.77 (b; 2H)		NH_2_
7.58 (dd, *J* = 8.1, 7.4 Hz; 1H)	*7.96*, 7.35, 7.30	H-8
7.37 (d, *J* = 0.8 Hz; 1H)		H-5′
7.35 (d, *J* = 8.3 Hz; 1H)	7.58	H-7
7.30 (dd, *J* = 7.8, 7.4 Hz; 1H)	7.96, 7.58	H-9
6.28 (d, *J* = 1.8 Hz; 1H)	*6.07*	H-3′
6.07 (dd, *J* = 2.0, 1.0 Hz; 1H)	*6.28*	H-4′
5.03 (s; 1H)		H-4
4.11 (**A**BX_3_, *J*_AB_ = 14.2, *J*_AX_ = 7.1 Hz; 1H)	1.21	H-3b
4.08 (A**B**X_3_, *J*_AB_ = 14.2, *J*_BX_ = 7.1 Hz; 1H)	1.21	H-3b
1.21 (AB**X**_**3**_, *J*_AX_ = *J*_BX_ = 7.1 Hz; 3H)	4.11, 4.08	H-3c

**Table:** 

^13^C NMR (DMSO-*d*_6_)	Assignment
167.7	C-3a
160.6, 160.1	C-2, C-2′
156.4	C-5
151.9	C-10b
141.0	C-5′
137.7	C-6a
131.0	C-8
121.9, 121.6	C-9, C-10
115.30	C-7
112.2, 110.2, 109.5	C-3′, C-4′, C-10a
105.0	C-4a
74.6	C-3
58.8	C-3b
28.2	C-4
14.3	C-3c

**Fig. 4:**
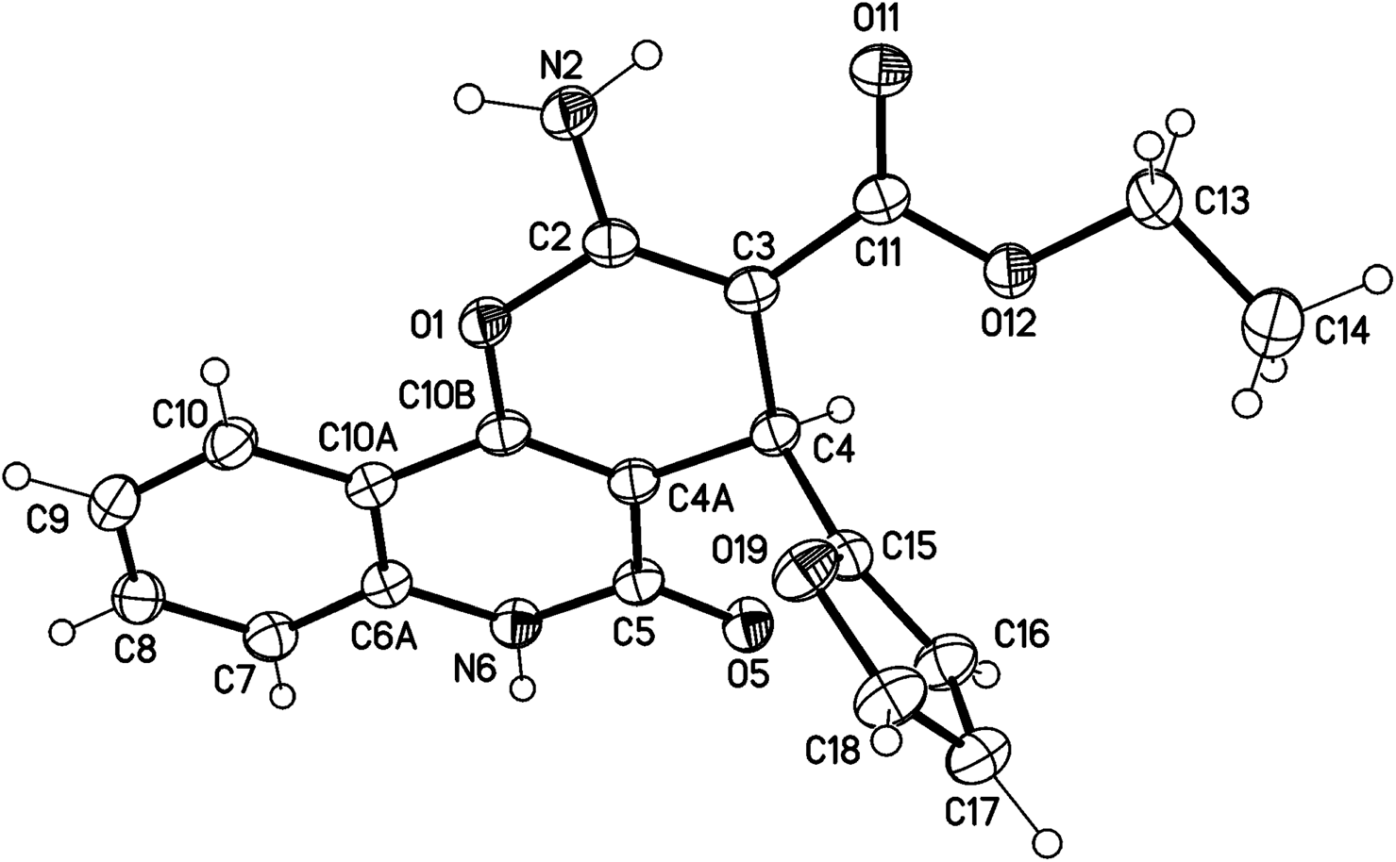
Molecular X-ray structure of compound 3a (displacement parameters are drawn at a 50% probability level).

X-ray structure analysis proved the structure of 3a (Fig. 4) and was identified as ethyl 2-amino-4-(furan-2-yl)-5-oxo-5,6-dihydro-4*H*-pyrano[3,2-*c*]quinoline-3-carboxylate.

**Fig. 5:**
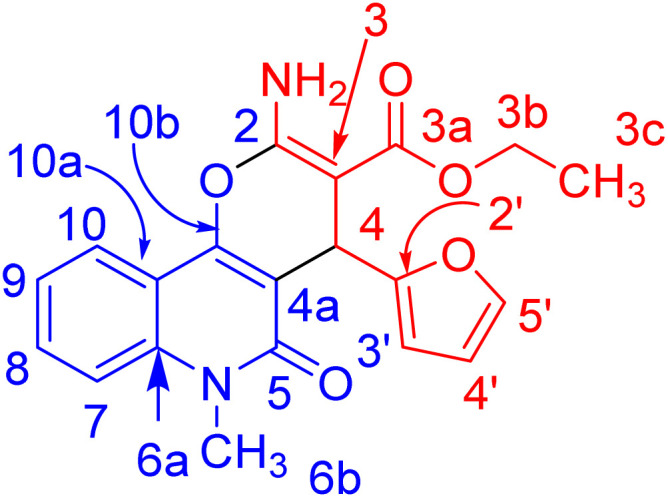
Distinctive carbons of compound 3b.

In compound 3b (Fig. 5), the ^1^H NMR spectrum revealed the CH_3_-ester as an AB**X**_3_, (*J*_AX_ = *J*_BX_ = 7.1 Hz) at *δ*_H_ = 1.21. The CH_2_ protons of the ester group appeared as non-equivalent two protons at *δ*_H_ = 4.12 (**A**BX_3_, *J*_AB_ = 10.7, *J*_AX_ = 7.1 Hz) and at *δ*_H_ = 4.06 ppm (**A**BX_3_, *J*_AB_ = 10.7, *J*_AX_ = 7.1 Hz). They have a COSY relationship with each other and with the CH_3_ protons of the ester group. The methyl (N–CH_3_) and CH-pyran (H-4) protons resonated at *δ*_H_ = 3.63 and 5.05 ppm. The three protons of furan appeared as a doublet at *δ*_H_ = 6.07 (*J* = 3.0 Hz) for H-4′ and a double-doublet at *δ*_H_ = 6.27 ppm (*J* = 3.0, 1.9 Hz) for H-3′. H-5′ of the furan molecule resonated as a doublet at *δ*_H_ = 7.36 (*J* = 0.8 Hz). The amino protons appeared in the ^1^H NMR spectrum at *δ*_H_ = 7.79. Table 3 illustrates the assigned *δ* values of the NMR spectral data for compound 3b.

Spectral data of NMR spectra of compound 3b

**Table:** 

^1^H NMR (DMSO-*d*_6_)	^1^H–^1^H COSY	Assignment
8.07 (dd, *J* = 7.9, 1.0 Hz; 1H)	*7.71*, 7.39, *6.07*	H-10
7.79 (b; 2H)		NH-2a
7.71 (ddd, *J* = 8.5, 7.2, 1.3 Hz; 1H)	*8.07*, 7.58, 7.39	H-8
7.58 (d, *J* = 8.5 Hz; 1H)	7.71, *7.39*	H-7
7.39 (dd, *J* = 7.7, 7.4 Hz; 1H)	8.07, 7.71, *7.58*	H-9
7.36 (d, *J* = 0.8 Hz; 1H)	6.27	H-5′
6.27 (dd, *J* = 3.0, 1.9 Hz; 1H)	7.36, 6.07	H-3′
6.07 (d, *J* = 3.0 Hz; 1H)	*8.07*, 6.27	H-4′
5.05 (s; 1H)		H-4
4.12 (**A**BX_3_, *J*_AB_ = 10.7, *J*_AX_ = 7.1 Hz; 1H)	4.06, 1.21	H-3b
4.06 (A**B**X_3_, *J*_AB_ = 10.7, *J*_BX_ = 7.1 Hz; 1H)	4.12, 1.21	H-3b
3.63 (s; 3H)		H-6b
1.21 (AB**X**_**3**_, *J*_AX_ = *J*_BX_ = 7.1 Hz; 3H)	4.12, 4.06	H-3c

**Table:** 

^13^C NMR (DMSO-*d*_6_)	Assignment
167.6	C-3a
160.0, 159.9	C-2, C-2′
156.4	C-5
150.9	C-10b
141.0	C-5′
138.5	C-6a
131.5	C-8
122.0	C-9, C-10
114.9	C-7
112.8, 110.2, 108.9	C-3′, C-4′, C-10a
105.1	C-4a
74.6	C-3
58.8	C-3b
29.3	C-6b
28.9	C-4
14.3	C-3c

**Fig. 6:**
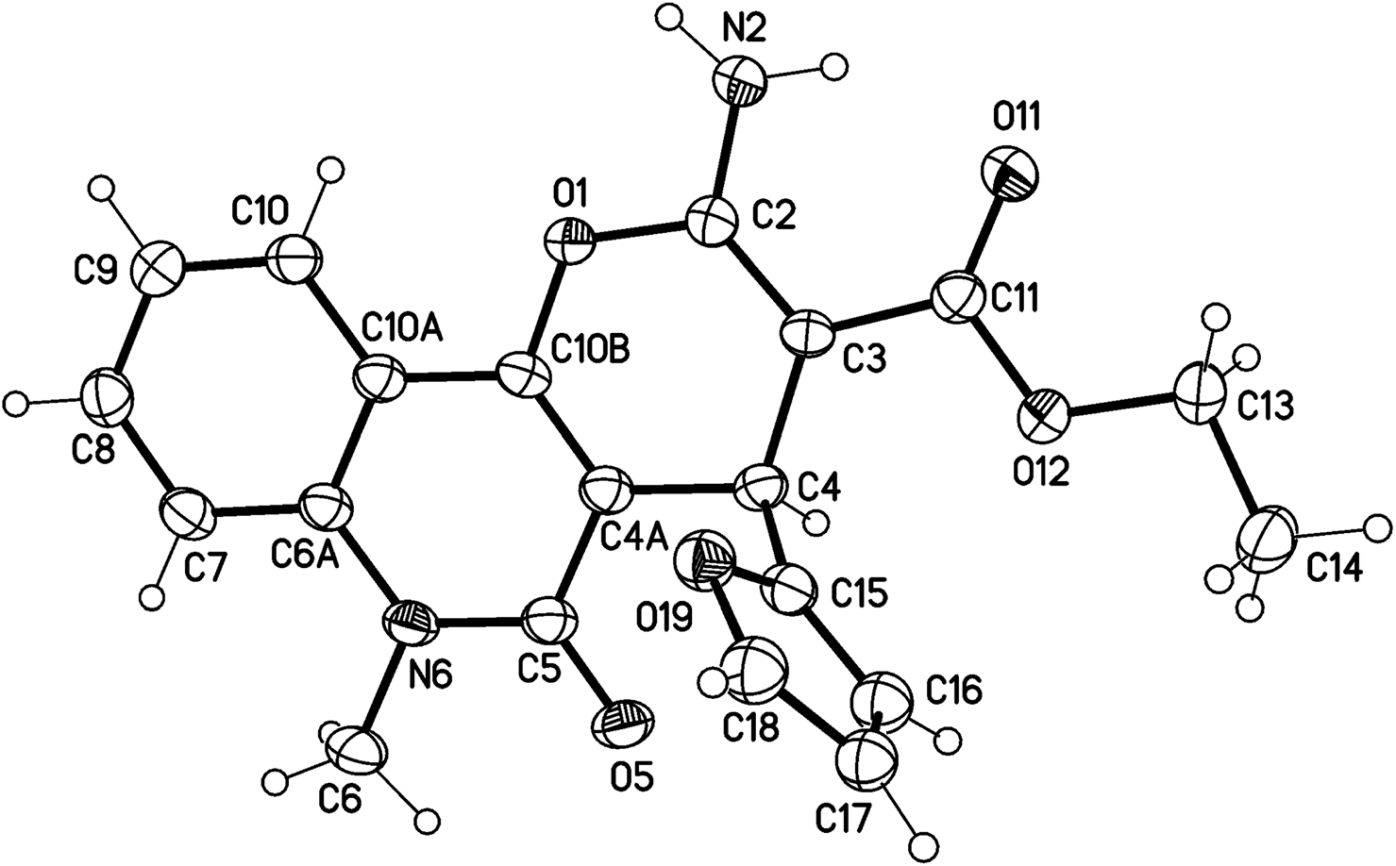
Molecular structure of the 1st crystallographic independent molecule of compound 3b (displacement parameters are drawn at a 50% probability level).

X-ray structure analysis proved the structure of 3b (Fig. 6) and was identified as ethyl 2-amino-4-(furan-2-yl)-6-methyl-5-oxo-5,6-dihydro-4*H*-pyrano [3,2-*c*]quinoline-3-carboxylate.

It is noteworthy that compounds 3a and 3b crystallized in a centrosymmetric space group (*P*1̄ (no. 2)) and the relative configuration was determined. Therefore, both enantiomers (*R* and *S* at C4 and C24, respectively) were present in a ratio of 1 : 1.

**Scheme 2:**
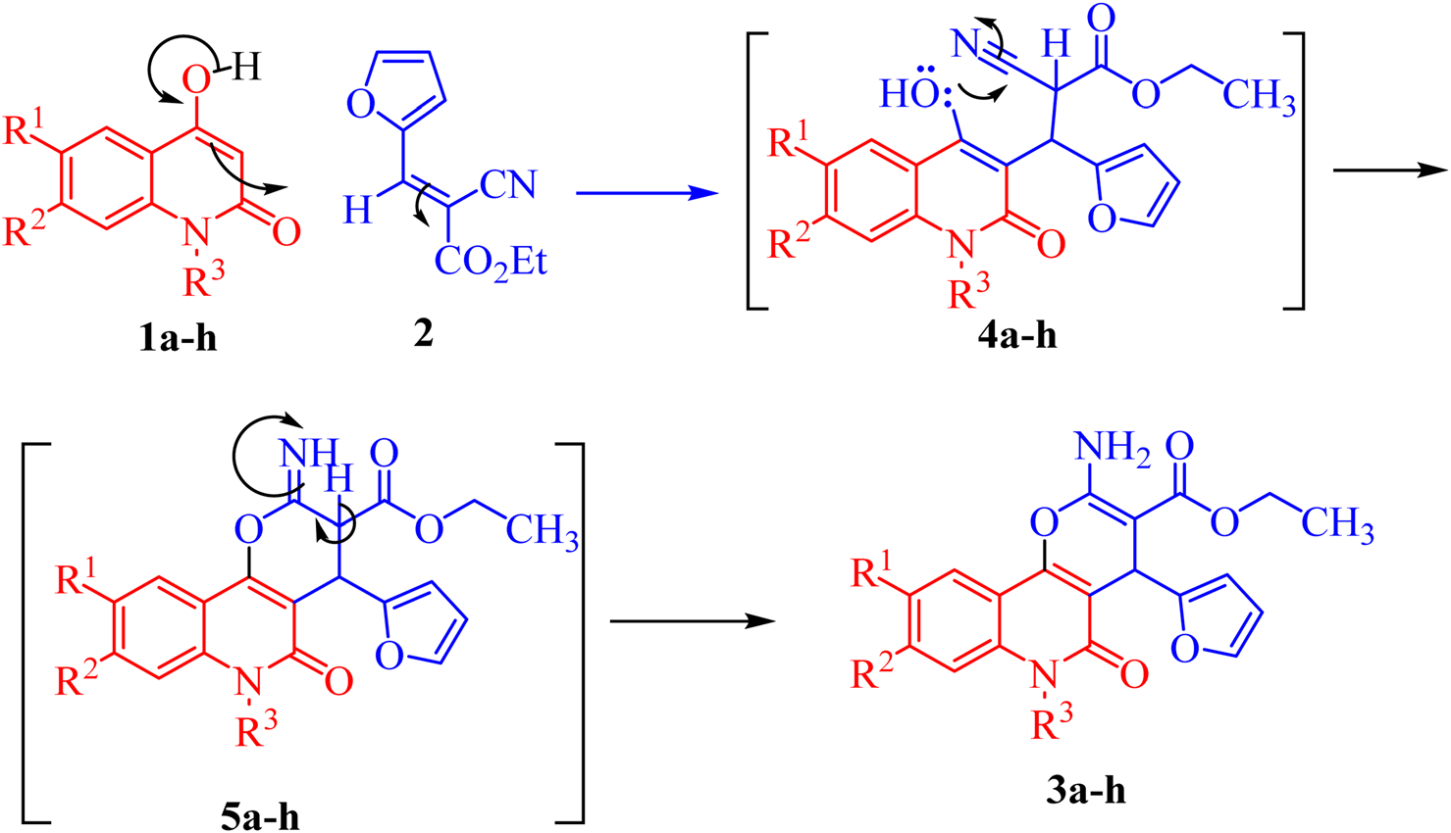
Mechanism describing the formation of compounds 3a–h.

The mechanism proposed for the formation of compounds 3a–h begins with a nucleophilic attack of the active CH-3 from 1a–h to the electrophilic carbon in 2*via* Michael addition to produce intermediates 4a–h (Scheme 2). Further nucleophilic attack of the hydroxyl-lone pair in 4a–h then occurs to the electrophilic carbon in the nitrile group, forming intermediate 5a–h (Scheme 2). Finally, the aromatization of 5a–h gives the final products 3a–h (Scheme 2).

### 2.2. Biological screening

#### 2.2.1. Cytotoxic activity

The newly synthesized compounds were evaluated owing to their *in vitro* antiproliferative activity against lung epithelial cancer cells A549 using the MTT viability assay with Levofloxacin as the standard drug (Table 4). Interestingly, compound 3h showed the highest antiproliferative activity with MIC equal to 35.10 μM, which is even greater than the positive control Levofloxacin 410.10 μM. Compounds 3d–g showed moderate activity among the series but were still more potent than the reference drug Levofloxacin. Compounds 3a–c exhibited the least potency among the designed compounds. SAR studies indicated that the halo-pyrano[3,2-*c*]quinoline-3-carboxylates clubbed compounds 3f–h were more potent inhibitors than the other non-halogenated derivatives 3a–e. Moreover, the pyrano quinolone derivative incorporating the bromo substituent (compound 3h) exhibited the highest antiproliferative activity against lung epithelial cancer cells A549. These results confirm that the incorporation of a halogen, especially a bromine atom, at the pyrano quinolone ring greatly enhanced the antiproliferative activity against lung epithelial cancer cells.

**Table 4:** IC_50_ of compounds 3a–h

Compound	Structures	A549	A549
		IC_50_ (μg mL^−1^)	IC_50_ (μM)
3a	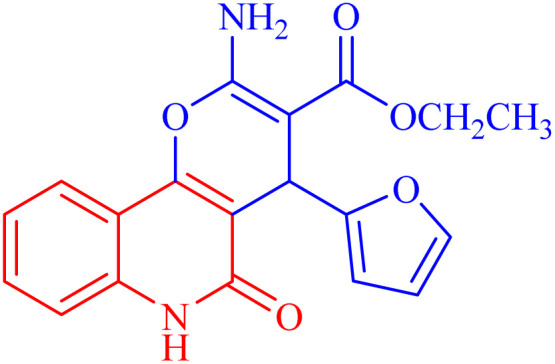	122.9 ± 3.6	348.8009
3b	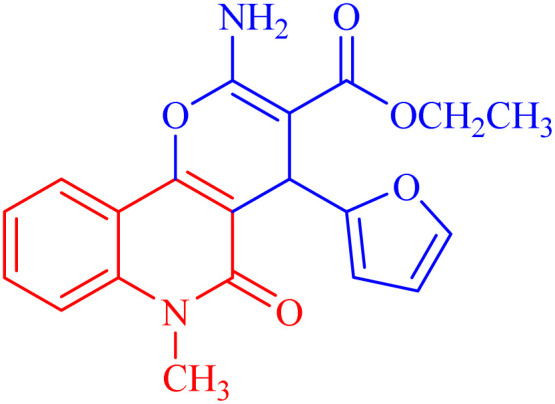	127.5 ± 1.1	440.8112
3c	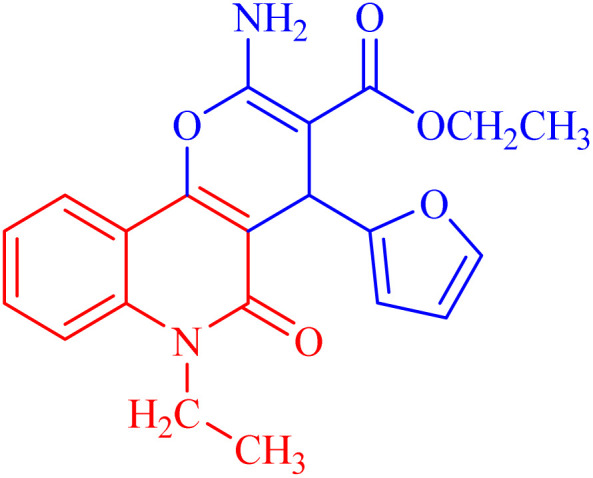	131.55 ± 3.2	417.9811
3d	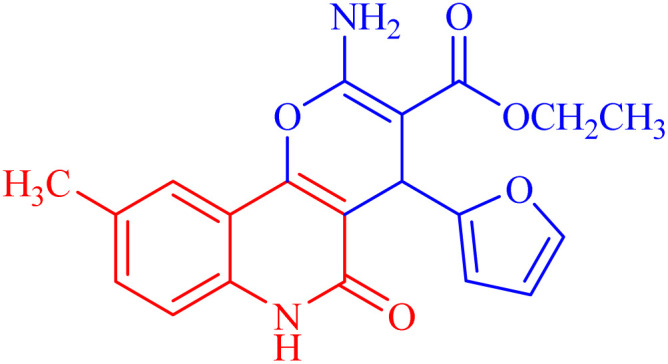	47.465 ± 3.06	116.194
3e	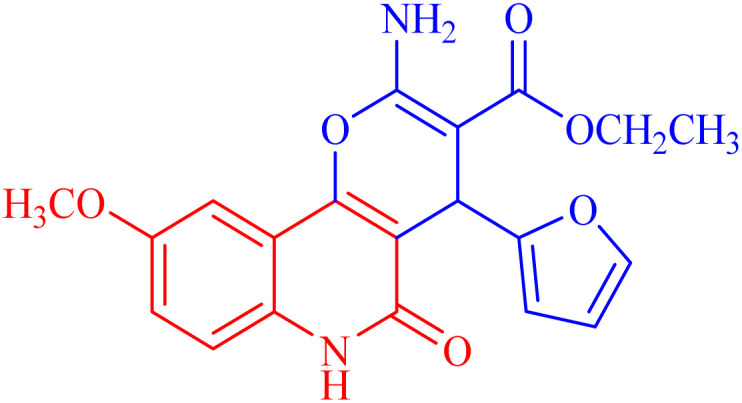	53.285 ± 0.815	139.3153
3f	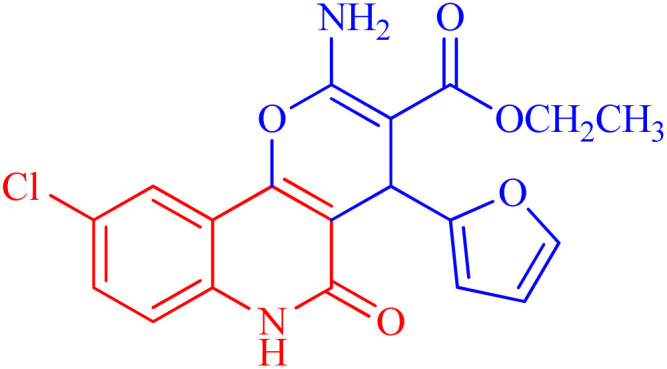	30.96 ± 1.47	78.56977
3g	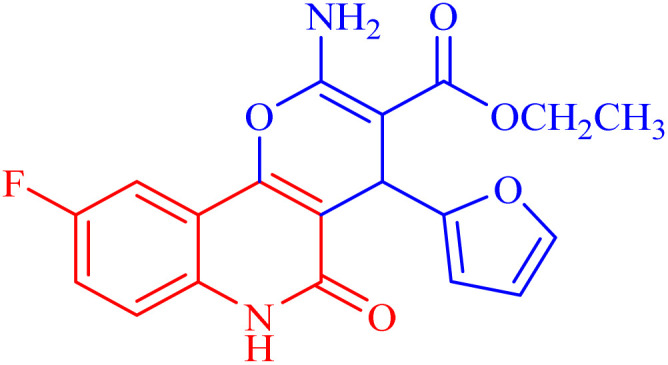	45.97 ± 0.87	125.6143
3h	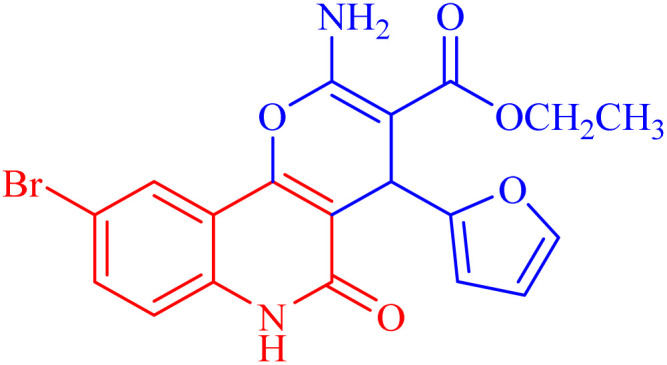	**15.64** ± 1.02	**35.10806**
Levofloxacin	—	148.2 ± 1.42	**410.10**

Besides considering cytotoxicity against A549 cells, testing safety on normal cells WI38 and selectivity to cancer cells is the main evaluation factor of the studied pyranoquinolones. Interestingly, the most active derivative 3h exhibited lower cytotoxic activity against normal cells (IC_50_ = 43.28 μM) with a selectivity index of 1.23.

#### 2.2.2. Topoisomerase II inhibition

The *in vitro* topoisomerase II inhibitory profile of the most potent derivative 3h was studied in reference to the standard etoposide. Compound 3h exhibited promising inhibitory activity with a micromolar IC_50_ value (IC_50_ = 45.19 μM), and it was slightly less potent than etoposide (IC_50_ = 34.99 μM) (Table 5).

**Table 5:** Inhibitory profile of 3h against topoisomerase II

Compound	Structure	IC_50_ (μM)
3h	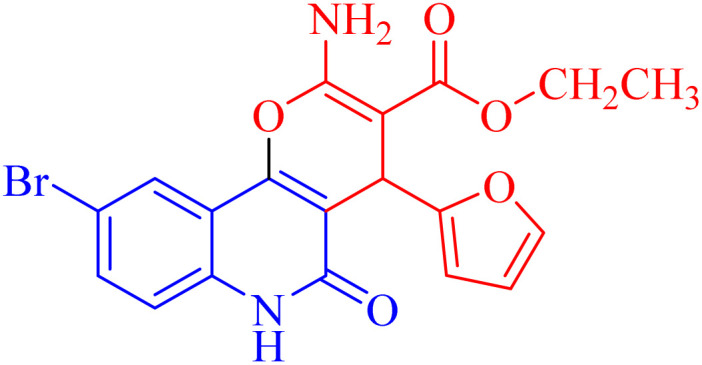	45.19 ± 3.37
**Etoposide**	—	34.99 ± 2.58

#### 2.2.3. Antibacterial activity

Biological evaluation for all the target compounds was initially performed using the agar diffusion method as a preliminary step to screen for the most potent compounds against *Staphylococcus aureus* ATCC6538 (Table 6). After screening, four compounds were selected because they demonstrated the highest antibacterial activity (higher inhibition zone diameter) towards the *Staphylococcus aureus* standard strain. These were further tested to determine their minimal inhibitory concentration (MIC) using the broth microdilution method according to the CLSI reference standard (Table 7) using Spiramycin as the standard drug. Compound 3h showed the highest activity among the whole series with MIC 90.58 μM. The designed derivative 3h exhibited potency comparable to the control drug Spiramycin with MIC 37.957 μM. SAR studies revealed that the halogenated pyranoquinolone derivatives 3f–h were more potent antibacterial agents than the other non-halogenated derivatives. The bromo-pyrano quinolone derivative (compound 3h) showed very promising antibacterial activity and exhibited the highest antibacterial activity against *Staphylococcus aureus*. These results indicate the great importance of the presence of a halogen, especially the electron withdrawing property of the bromine atom.

**Table 6:** Inhibition zone diameter of compounds 3a–h

Compound	Inhibition zone diameter (mm) DMSO control (8 mm)
3a	8
3b	8
3c	8
3d	10 ± 0.5
3e	9 ± 0.3
3f	17.5 ± 0.85
3g	18 ± 1
3h	19 ± 1.1

**Table 7:** Minimal inhibitory concentration of compounds 3f–h

Compound	MIC (μg mL^−1^)	MIC (μM)
3f	78.125	201.983
3g	78.125	210.954
3h	**39.062**	**90.580**
**Spiramycin** ^30^	**32**	**37.957**

#### 2.2.4. Bacterial DNA gyrase inhibition

DNA gyrase inhibition assay (Table 8) showed that 3h exhibited significant potency against DNA gyrase, and it recorded % inhibition comparable to the reference Levofloxacin. Further investigations indicated that 3h exhibited micromolar IC_50_ (IC_50_ = 40.76 μM) relative to the reference inhibitor.

**Table 8:** Inhibitory profile of 3h against DNA gyrase

Compound	Structure	IC_50_ (μM)
3h	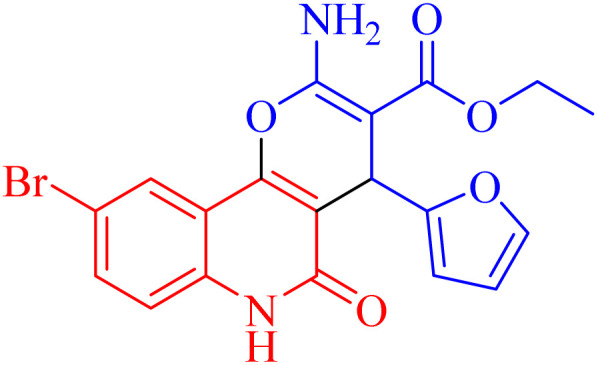	40.76 ± 0.64
Levofloxacin	—	31.34 ± 0.45

#### 2.2.5. Apoptotic investigation and cell cycle analysis

**Fig. 7:**
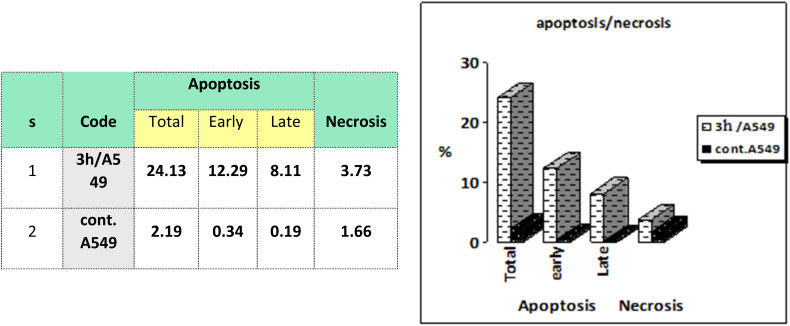
Representation of A549 cells treated with 3h and analyzed using flow cytometry after double staining of the cells with annexin-V FITC and PI.

Flow cytometric analysis of annexin V/PI staining was performed to determine the apoptotic cell death compared to the necrotic one to investigate the apoptotic activity of the tested compound 3h (IC50 = 35.10 μM), in the untreated and treated lung epithelial cancer cells A549. As shown in Fig. 7, compound 3h significantly stimulated apoptotic lung cancer cell death by 38.49-fold; it increased total apoptosis by 20.4% (8.11% for late and 12.29% for early) compared to 0.53% (0.19% for late and 0.34% for early) for the control. On the contrary, the compound stimulated necrotic lung cancer cell death by 2.24-fold; it induced necrotic cell death by 3.73% compared to 1.66%. Therefore, the compound treatment favors apoptotic cell death rather than necrosis.

**Fig. 8:**
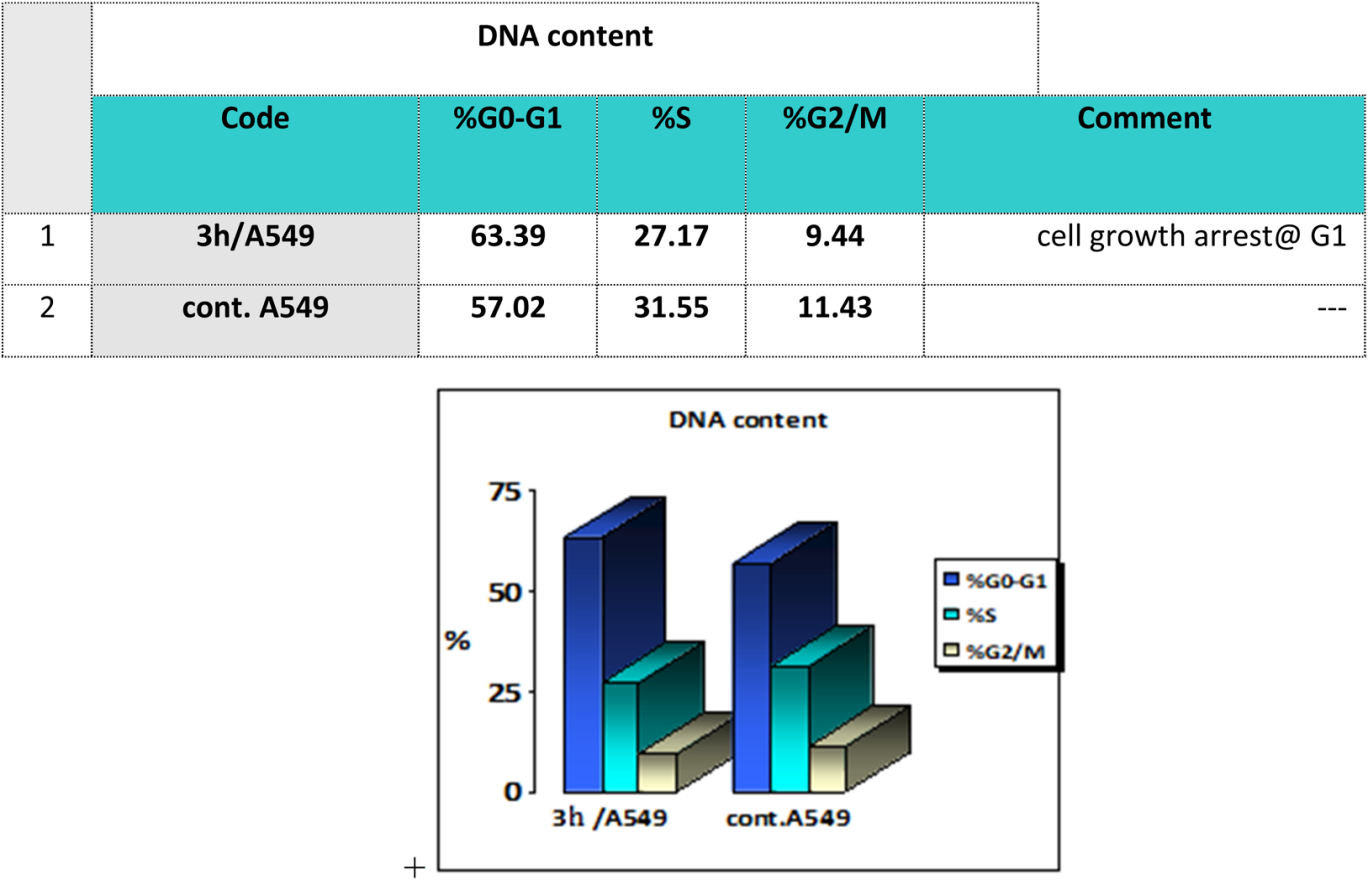
Bar representation of the percentage of cell population at each cell cycle G1, S and G2/M.

Cell cycle analysis is a crucial test that investigates the percentages of the cell population in each cell phase with cytotoxic substances after treatment. Lung epithelial cancer cells A549 were treated with compound 3h. It was subjected to DNA flow cytometry to determine at which cell cycle the cell proliferation was arrested. As shown in Fig. 8, the compound treatment significantly increased the cell population at the G1 phase by 63.39% compared to the control 57.02%. In comparison, the other phases did not significantly change.

### 2.3. Molecular docking

A computational study utilizing MOE 2019.10259 was employed to predict the binding scores and modes of the investigated derivatives 3a–h into the target proteins topoisomerase II (PDB: 5GWK^31^) and DNA gyrase (PDB: 2XCT^32^) compared to co-crystallized ligands etoposide and ciprofloxacin, respectively.

#### 2.3.1. Topoisomerase II

**Fig. 9:**
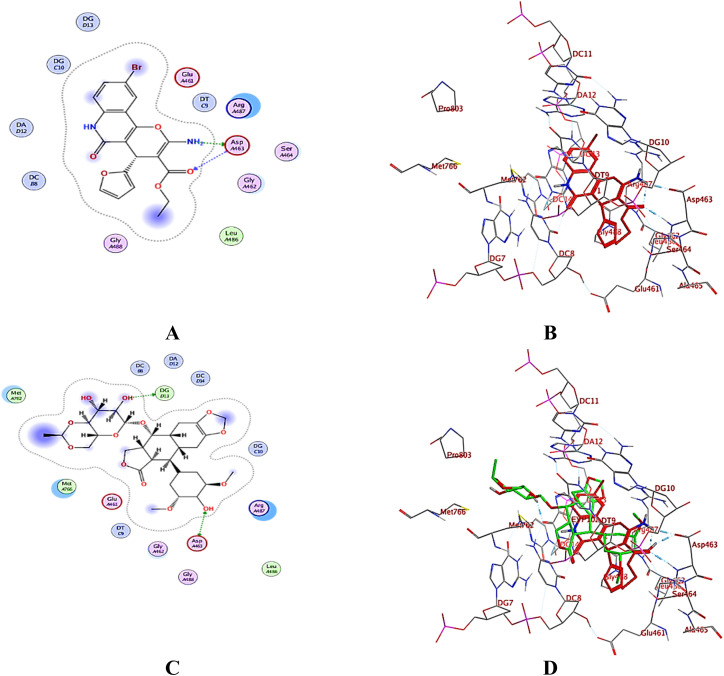
(A) 2D-binding mode of 3h, (B) 3D-interactions of 3h (red sticks), (C) 2D-binding mode of the co-crystallized ligand etoposide, and (D) 3D-overlay of 3h (red sticks) and etoposide (green sticks) (PDB: 5GWK^31^).

Test compounds fitted well in the co-crystalized ligand binding site with promising binding scores ranging from −7.55 to −8.19 kcal mol^−1^ (ESI Data†), compared to the redocked ligand etoposide (binding score = −11.02 at RMSD = 0.35 Å). As illustrated in Fig. 9A and B, 3h interacted with the essential amino acid residue Asp 463 through hydrogen bonds, which can be compared to etoposide. However, the remaining favorable compounds (ESI Data†) were able to dock deeply within the enzyme pocket near the DNA, forming different types of interactions, including hydrogen bonding and hydrophobic interactions, with both the protein and DNA.

#### 2.3.2. DNA gyrase

**Fig. 10:**
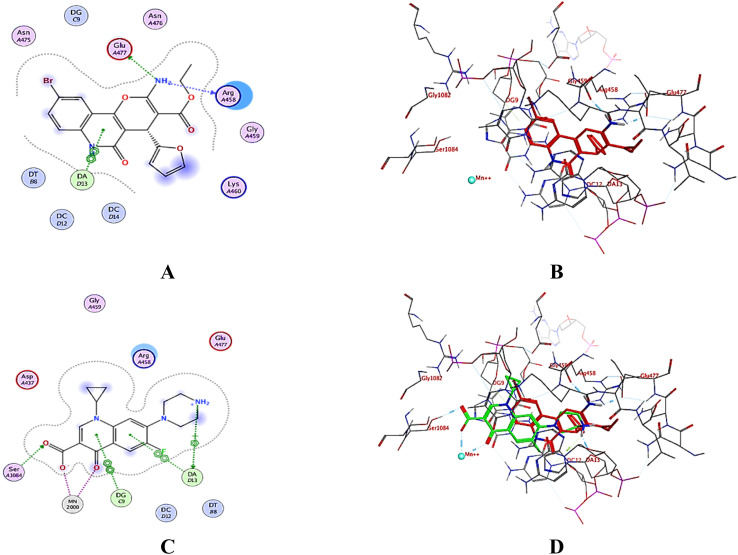
(A) 2D-binding mode of 3h, (B) 3D-interactions of 3h (red sticks), and (C) 2D-binding mode of the co-crystallized ligand ciprofloxacin, and (D) 3D-overlay of 3h (red sticks) and ciprofloxacin (green sticks) (PDB: 2XCT^32^).

Validation was done by redocking the co-crystallized ligand ciprofloxacin into the enzyme active site to ensure that its experimental interactions were reproduced (docking score = −9.92 kcal mol^−1^ at RMSD = 0.64 Å). Ciprofloxacin showed a hydrogen bond with Ser1084, and it was also bonded with DNA *via* π interactions with the guanine DG:C9 and adenine bases DA:D13 (Fig. 10). Interestingly, the tested compounds displayed relatively high docking scores ranging from −8.03 to −8.63 kcal mol^−1^ compared to ciprofloxacin. Additionally, they showed nearly similar binding modes (ESI Data†). Compound 3h showed the best binding score (−8.63 kcal mol^−1^) and resided well into the active site through π interaction with the DNA adenine base DA:D13 in addition to hydrogen bonds with Arg458 and Glu477 (Fig. 10). These observations assumed that screened compounds can be potential antibacterial agents targeting DNA gyrase as the docking results are generally consistent with the *in vitro* antibacterial activities, where 3h was superior to the remaining compounds.

### *2.4*. *In silico* drug likeness and molecular property prediction

Molecular property prediction is becoming a useful tool in the generation of molecules with the correct parameters to be useful drug candidates. About 40% of oral drugs fail in clinical trials because of their poor pharmacokinetic properties. Drug development involves the assessment of absorption, distribution, metabolism and excretion (ADME) increasingly earlier in the discovery process, at a stage when the considered compounds are numerous, but access to physical samples is limited. Thus, we applied computational methods to predict physical and molecular properties to assess their availability as useful drug candidates. Here, we present a new SwissADME web tool that provides free access to a pool of fast yet robust predictive models for physicochemical properties, pharmacokinetics, drug-likeness and medicinal chemistry friendliness, including in-house proficient methods such as iLOGP and Bioavailability Radar (Table 9).

**Table 9:** ADME analysis of compounds 3a–h

ID	*M* a	Log *P*^b^	HBD^c^	HBA^d^	Nrotb^e^	TPSA^f^	MR^g^	Log *S*^h^	*F* i	GI absorption	BBB^j^	Pgp^k^ substrate
3a	352.34	2.44	2	5	4	107.55	93.41	−3.47	0.56	High	No	No
3b	366.37	3.15	1	5	4	96.69	82.02	−2.31	0.56	High	No	No
3c	380.39	3.38	1	5	5	96.69	103.12	−3.89	0.56	High	No	No
3d	366.37	3.04	2	5	4	107.55	98.38	−3.77	0.56	High	No	Yes
3e	382.37	2.75	2	6	5	116.78	99.9	−3.54	0.56	High	No	Yes
3f	386.79	2.75	2	5	4	107.55	98.42	−4.06	0.56	High	No	No
3g	370.33	2.81	2	6	4	107.55	93.37	−4.03	0.56	High	No	No
3h	431.24	3.17	2	5	4	107.55	101.11	−4.38	0.56	High	No	No

a *M*, molecular weight (dalton).

b *i* log *P*, octanol/water partition coefficient.

c HBD, hydrogen bond donor.

d HBA, hydrogen bond acceptor.

e Nrotb, # of rotatable bonds.

f TPSA, total polar surface area.

g MR, molar refractivity.

h *i* log *P*, logarithm of compound aqueous solubility.

i *F*, Abbott oral bioavailability score HIA%, human gastrointestinal absorption.

j BBB permeant, blood–brain barrier penetration.

k Pgp, permeability glycoprotein.

All the compounds had high GI absorption and obeyed the Lipinski rule of five. The overall results showed that the tested compound showed a very good drug-likeness pharmacokinetic and pharmacodynamic profile.

## 3. Conclusion

A series of new pyrano[3,2-*c*]quinoline-3-carboxylate derivatives 3a–h were designed and synthesized efficiently using a microwave. The structure of the products was confirmed using a combination of spectral techniques, including infra-red (IR), nuclear magnetic resonance (NMR), mass spectrometry (MS) and elemental analyses, in addition to X-ray structure analysis. The newly synthesized compounds were evaluated for their *in vitro* antiproliferative activity against lung epithelial cancer cells A549 using an MTT viability assay. Compound 3h showed the highest activity with an MIC of 15.14 μg ml^−1^ compared to the positive control Levofloxacin. The cell cycle arrest behavior detected by propidium iodide and the apoptosis induction was investigated. In addition, the newly synthesized compounds were evaluated for their *in vitro* antibacterial activity against the standard *Staphylococcus aureus* strain. Compound 3h showed the highest antibacterial activity with an MIC of 39.062 μg ml^−1^ using Levofloxacin as the standard reference drug. An *in silico* study was performed, including docking of the newly synthesized compounds into topoisomerase II and DNA-gyrase binding pockets, in addition to predicting their physicochemical and pharmacokinetic properties. *In silico* studies showed the ability of the synthesized members to bind to the topoisomerase II and DNA-gyrase. Our study revealed that compound 3h is a promising dual acting anticancer and antibacterial agent targeting topoisomerase II and DNA-gyrase with acceptable oral bioavailability and physicochemical and pharmacokinetic properties.

## 4. Experimental

### 4.1. Chemistry

Melting points were measured in open capillaries using a Gallenkamp melting point apparatus (Weiss-Gallenkamp, Loughborough, UK) and were uncorrected. The IR spectra were recorded by applying the ATR technique (ATR = Attenuated Total Reflection) with an FT device (FT-IR Bruker IFS 88), Institute of Organic Chemistry, Karlsruhe University, Karlsruhe, Germany. The NMR spectra were measured in DMSO-*d*_6_ using a Bruker AV-400 spectrometer, 400 MHz for ^1^H, and 100 MHz for ^13^C. The chemical shifts are expressed in *δ* (ppm) *versus* internal tetramethylsilane (TMS) = 0 for ^1^H and ^13^C. The description of signals includes s = singlet, d = doublet, dd = doublet of doublet, t = triplet, q = quartet, and m = multiplet. The following abbreviations were used to distinguish between signals: Ar-H = aromatic-CH. Signals of the ^13^C NMR spectra were assigned with the help of DEPT90 and DEPT135 and were specified in the following way: + = primary or tertiary carbon atoms (positive DEPT signal), − = secondary carbon atoms (negative DEPT signal), and C_q_ = quaternary carbon atoms (no DEPT signal). Correlations were established using ^1^H–^1^H COSY, ^1^H–^13^C HSQC and HMBC experiments. Mass spectra were recorded using a FAB (fast atom bombardment) Thermo Finnigan Mat 95 (70 eV). The HRSM was recorded using LC/Q-TOF, 6530 (Aligent Technologies, Santa Clara, CA, USA) at the Natural Product Research lab, Faculty of Pharmacy, Fayoum University, Egypt. Elemental analyses were carried out at the Microanalytical Center, Cairo University, Egypt. TLC was performed on analytical Merck 9385 silica aluminum sheets (Kieselgel 60) with Pf254 indicator; TLCs were viewed at *λ*_max_ = 254 nm.

#### 4.1.1. Starting materials

1,6-Disubstituted-quinoline-2,4-(1*H*,3*H*)-diones 1a–h were prepared according to the literature.^33,34^ Ethyl (*E*)-2-cyano-3-(furan-2-yl)acrylate (2) was purchased from Aldrich (St. Louis, MO, USA).

#### 4.1.2. Reactions of 1a–h with 2; synthesis of compounds 3a–h

##### 4.1.2.1. Method I

A mixture of 1a–h (1 mmol) and 2 (1 mmol, 0.138 g) in 40 ml absolute ethanol and anhydrous K_2_CO_3_ (1.5 mmol, 0.192 g) was stirred at room temperature for 12–24 h. The reaction was monitored through TLC analysis. The formed products 3a–h were filtered off and washed several times with H_2_O (150 ml) and with ether (30 ml). The obtained products were recrystallized from the stated solvents to afford pure compounds 3a–h.

##### 4.1.2.2. Method II

The above method was repeated by exposing the reaction mixture to MW irradiation for 3–10 min. The reaction was refluxed in a Milestone Microwave Lab station at 120 °C for 3–10 min. All reactions were monitored by TLC with 1 : 1 ethyl acetate/petroleum ether as an eluent and were carried out until the starting materials were completely consumed. After few minutes (Table 1), microwave irradiation was stopped, and the reaction mixture was analyzed by TLC.

###### 4.1.2.2.1 Ethyl 2-amino-4-(furan-2-yl)-5-oxo-5,6-dihydro-4*H*-pyrano[3,2-*c*]quinoline-3-carboxylate (3a)

Compound 3a was obtained as pale brown crystals, (DMF/EtOH); yield: m.p. 270–272 °C; IR (KBr): *ν*_max_/cm^−1^ = 3460–3463 (NH_2_), 3325 (NH), 3100 (CH-Ar), 2975 (CH-Aliph), 1692 (C=O, ester), 1656 (C=O, quinolone), 1578 (C=C), 1437 (CH_2_), 1375 (CH_3_). NMR (see Table 2). MS (FAB, 3-NBA), *m*/*z* (%): 352.1 [M^+^] (18), 353.1 [M + 1] (22), 307.1 (100), 289.1 (35). Anal. calcd C_19_H_16_N_2_O_5_ (352.35): C, 64.77; H, 4.58; N, 7.95. Found: C, 64.85; H, 4.48; N, 8.10.

###### 4.1.2.2.2 Ethyl 2-amino-4-(furan-2-yl)-6-methyl-5-oxo-5,6-dihydro-4*H*-pyrano[3,2-*c*]quinoline-3-carboxylate (3b)

Compound 3b was obtained as pale brown crystals, (DMF/EtOH); yield: 92%; m.p. 260–262 °C. IR (KBr): *ν*_max_/cm^−1^ = 3275 (NH), 3050 (CH-Ar), 2928 (CH-Aliph), 1682 (C=O, ester), 1654 (C=O, quinolone), 1614 (C=C), 1463 (CH_2_), 1418 (N–CH_3_), 1375 (CH_3_). NMR (see Table 3). MS (FAB, 3-NBA), *m*/*z* (%): 366.1 [M]^+^ (35), 367.1 [M + 1] (45). HRSM [M + Na^+^] = calcd: 389.1114; found: 389.1124. Anal. calcd for C_20_H_18_N_2_O_5_ (366.37): C, 65.57; H, 4.95; N, 7.65. Found: C, 65.47; H, 4.85; N, 7.70.

###### 4.1.2.2.3 Ethyl 2-amino-6-ethyl-4-(furan-2-yl)-5-oxo-5,6-dihydro-4*H*-pyrano[3,2-*c*]quinoline-3-carboxylate (3c)

Compound 3c was obtained as pale brown crystals, (DMF/EtOH), yield: 94%; m.p. 264–266 °C. IR (KBr): *ν*_max_/cm^−1^ = 3268 (NH), 3056 (CH-Ar), 2828 (CH-Aliph), 1767 (C=O, pyranone), 1655 (C=O, ester), 1541 (C=O, quinolone), 1485 (CH_2_), 1375 (CH_3_). ^1^H NMR (DMSO-*d*_6_, ppm): *δ*_H_ = 8.08 (dd, *J* = 7.9, 0.8 Hz, 1H; H-10), 7.76 (b, 2H; NH-2a), 7.64 (ddd, *J* = 8.3, 7.0, 1.00 Hz, 1H; H-8), 7.57 (d, *J* = 8.4 Hz, 1H; H-7), 7.34 (dd, *J* = 8.3, 7.5 Hz, 1H; H-9), 7.33 (bs; H-5′), 6.26 (dd, *J* = 3.0, 1.9 Hz, 1H; H-3′), 6.03 (d, *J* = 3.0 Hz, 1H; H-4′), 5.06 (s, 1H; H-4), 4.34–4.22 (m, 4H, 2×C*H*_2_), 1.27 (t, *J* = 7.1 Hz, 3H, H-3c), 1.20 (t, *J* = 7.0 Hz, 3H, H-6c). ^13^C NMR (DMSO-*d*_6_, ppm): *δ*_C_ = 167.6 (C-3a), 159.9, 159.9 (C-2, C-2′), 156.3 (C-5), 150.9 (C-10b), 141.0 (C-5′), 138.6 (C-6a), 131.4 (C-8), 122.1 (C-9, C-10), 114.9 (C-7), 112.8, 110.3, 108.9 (C-3′, C-4′, C-10a), 105.1 (C-4a), 74.6 (C-3), 58.9 (C-3b), 38.4 (C-6b), 28.9 (C-4), 14.3 (C-3c) 12.6 (C-6c). MS (FAB, 3-NBA), *m*/*z* (%): 379.06 [M^+^ − 1] (35), 382.54 [M + 2]^+^ (18), 267.07 (100). HRSM [M + Na^+^] = calcd: 403.1270; found: 403.1281. Anal. calcd for C_21_H_20_N_2_O_5_ (380.40): C, 66.31; H, 5.30; N, 7.36. Found: C, 66.35; H, 5.28; N, 7.46.

###### 4.1.2.2.4 Ethyl 2-amino-4-(furan-2-yl)-9-methyl-5-oxo-5,6-dihydro-4*H*-pyrano[3,2-*c*]quinoline-3-carboxylate (3d)

Compound 3d was obtained as brown crystals (DMF); yield; 92%; m.p. 280–282 °C. IR (KBr): *ν*_max_/cm^−1^ = 3482–3435 (NH_2_), 3266 (NH), 3090 (CH-Ar), 2840 (CH-Aliph), 1737 (C=O, pyranone), 1695 (C=O, ester), 1521 (C=O, quinolone), 1415 (CH_2_), 1325 (CH_3_). ^1^H NMR (DMSO-*d*_6_, ppm): *δ*_H_ = 11.73 (s, 1H, NH), 7.96 (s, 1H; H-10), 7.77 (b, 2H, NH_2_), 7.58 (d, *J* = 8.1, 7.3 Hz, 1H, H-8), 7.35 (d, *J* = 0.8 Hz, 1H, H-5′), 7.38 (d, *J* = 8.2 Hz, 1H, H-7), 6.25 (d, *J* = 1.8 Hz, 1H, H-3′), 6.02 (d, *J* = 2.0 Hz, 1.0 Hz, 1H, H-4′), 5.06 (s, 1H, H-4), 4.09 (q, *J* = 7.05 Hz, 2H, H-3b), 2.24 (s, 3H; H-9a), 1.20 (t, *J* = 7.06 Hz, 3H, H-3c). ^13^C NMR (DMSO-*d*_6_, ppm): *δ*_C_ = 167.6 (C-3a), 160.6, 159.1 (C-2, C-2′), 156.3 (C-5), 151.9 (C-10b), 141.1(C-5′), 137.6 (C-6a), 131.0 (C-9), 122.6, 122.6 (C-8, C-10), 115.4 (C-7), 112.2, 110.2, 109.5 (C-3′, C-4′, C-10a), 105.0 (C-4a), 74.7 (C-3), 58.9 (C-3b), 28.22 (C-4), 20.3 (C-9a), 14.3 (C-3c). MS (FAB, 3-NBA), *m*/*z* (%): 366.46 [M]^+^ (50), 293.25 [M]^+^ (100), 253.22 (100), 224.24 (100). HRMS [M + Na^+^] = calcd: 389.1114; found: 389.1121. Anal. calcd for C_20_H_18_N_2_O_5_ (366.37): C, 65.57; H, 4.95; N, 7.65. Found: C, 64.57; H, 4.85; N, 7.61.

###### 4.1.2.2.5 Ethyl 2-amino-4-(furan-2-yl)-9-methoxy-5-oxo-5,6-dihydro-4*H*-pyrano[3,2-*c*]quinoline-3-carboxylate (3e)

Compound 3e was obtained as brown crystals, (DMF/EtOH); yield: 88%; m.p. 390–292 °C. IR (KBr): *ν*_max_/cm^−1^ = 3432–3475 (NH_2_), 3268 (NH), 3056 (CH-Ar), 2828 (CH-Aliph), 1767 (C=O, pyranone), 1655 (C=O, ester), 1541 (C=O, quinolone), 1485 (CH_2_), 1375 (CH_3_). ^1^H NMR (DMSO-*d*_6_, ppm): *δ*_H_ = 12.23 (s, 1H, NH), 7.98 (s, 1H; H-10), 7.79 (b, 2H, NH_2_), 7.61 (d, *J* = 8.2 Hz, 7.4, 1H, H-8), 7.38 (d, *J* = 0.7 Hz, 1H, H-5′), 7.41 (d, *J* = 8.0 Hz, 1H, H-7), 6.27 (d, *J* = 1.8 Hz, 1H, H-3′), 6.05 (d, *J* = 2.1 Hz, 1.0, 1H, H-4′), 5.04 (s, 1H, H-4), 4.06 (q, *J* = 7.0 Hz, 2H, H-3b), 3.71 (s, 3H; H-9a), 1.18 (t, *J* = 7.0 Hz, 3H, H-3c).^13^C NMR (DMSO-*d*_6_, ppm): *δ*_C_ = 167.1 (C-3a), 161.6, 161.5 (C-2, C-5), 158.3 (C-9, C-10b), 154.9 (C-2′), 145.7 (C-5′), 135.0 (C-6a), 124.7 (C-7), 118.0 (C-10a), 114.9, 112.7, 110.7 (C-8, C-10, C-4′), 107.8 (C-3′), 103.1 (C-4a), 75.7 (C-3), 61.3 (C-3b), 56.9 (C-9a), 28.2 (C-4), 14.6 (C-3c). MS (FAB, 3-NBA), *m*/*z* (%): 382.15 [M + 1] (30), 175.93 [M]^+^ (45). HRMS [M + H^+^] = calcd: 383.1243; found: 383.1227. Anal. calcd for C_20_H_18_N_2_O_6_ (382.37): C, 62.82; H, 4.75; N, 7.33. Found: C, 62.72; H, 4.55; N, 7.53.

###### 4.1.2.2.6 Ethyl 2-amino-9-chloro-4-(furan-2-yl)-5-oxo-5,6-dihydro-4*H*-pyrano[3,2-*c*]quinoline-3-carboxylate (3f)

Compound 3f was obtained as brown crystals, (DMF/CH_3_OH); yield: 80%; m.p. 310–312 °C. IR (KBr): *ν*_max_/cm^−1^ = 3432–3475 (NH_2_), 3222 (NH), 3010 (CH-Ar), 2812 (CH-Aliph), 1761 (C=O, pyranone), 1650 (C=O, ester), 1540 (C=O, quinolone), 1475 (CH_2_), 1335 (CH_3_). ^1^H NMR (DMSO-*d*_6_, ppm): *δ*_H_ = 12.42 (s, 1H, NH), 7.98 (d, *J* = 8.1 Hz, 1H, H-7), 7.81 (b, 2H, NH_2_), 7.41 (s, 1H, H-10), 7.37 (d, *J* = 0.9 Hz, 1H, H-5′), 7.32 (d, *J* = 8.1 Hz, 1H, H-8), 6.25 (d, *J* = 1.7 Hz, 1H, H-3′), 6.01 (d, *J* = 2.0 Hz, 1.0, 1H, H-4′), 5.02 (s, 1H, H-4), 4.02 (q, *J* = 7.04 Hz, 2H, H-3b), 1.16 (t, *J* = 7.03 Hz, 3H, H-3c). ^13^C NMR (DMSO-d_6_, ppm): *δ*_C_ = 167.9 (C-3a), 160.5, 160.3 (C-2, C-2′), 158.4(C-5), 155.0 (C-10b), 142.7 (C-5′), 135.2 (C-6a), 131.2 (C-9), 128.9, 126.6 (C-8, C-10), 122.6 (C-7), 114.2, 110.7, 108.7 (C-10a, C-4′, C-3′), 100.6(C-4a), 75.6 (C-3), 61.3 (C-3b), 28.5(C-4), 14.1 (C-3c). MS (FAB, 3-NBA), *m*/*z* (%): 386.07 [M + 1] (10), 366.57 [M]^+^ (15). HRMS [M + H^+^] = calcd: 387.0747; found: 387.0617. Anal. calcd for C_19_H_15_ClN_2_O_5_ (386.79): C, 59.00; H, 3.91; Cl, 9.17; N, 7.24. Found: C, 59.08; H, 3.95; Cl, 9.21; N, 7.30.

###### 4.1.2.2.7 Ethyl 2-amino-9-fluoro-4-(furan-2-yl)-5-oxo-5,6-dihydro-4*H*-pyrano[3,2-*c*]quinoline-3-carboxylate (3g)

Compound 3g was obtained as pale brown crystals, (DMF/EtOH); yield: 78%; m.p. 302–304 °C. IR (KBr): *ν*_max_/cm^−1^ = 3442–3455 (NH_2_), 3261 (NH), 3052 (CH-Ar), 2820 (CH-Aliph), 1760 (C=O, pyranone), 1659 (C=O, ester), 1549 (C=O, quinolone), 1445 (CH_2_), 1355 (CH_3_). ^1^H NMR (DMSO-*d*_6_, ppm): *δ*_H_ = 12.45 (s, 1H, NH), 7.88 (d, *J* = 8.2 Hz, 1H, H-7), 7.81 (b, 2H, NH_2_), 7.37 (d, *J* = 0.8 Hz, 1H, H-5′), 7.20 (d, *J* = 8.2 Hz, 1H, H-8) 7.02 (s, 1H, H-10), 6.27 (d, *J* = 1.7 Hz, 1H, H-3′), 6.02 (d, *J* = 2.01 Hz, 1.0, 1H, H-4′), 5.04 (s, 1H, H-4), 4.03 (q, *J* = 7.01 Hz, 2H, H-3b), 1.14 (t, *J* = 7.01 Hz, 3H, H-3c). ^13^C NMR (DMSO-*d*_6_, ppm): *δ*_C_ = 167.6 (C-3a), 160.6, 160.1 (C-2, C-9), 156.3 (C-5), 152.9, 152.6 (C-10b, C-2′), 141.9 (C-5′), 135.7 (C-6a), 118.0 (C-10a), 115.7, (C-8), 112.6 (C-7), 111.4, 111.3 (C-10, C-4′), 106.0 (C-3′), 100.9 (C-4a), 74.7 (C-3), 59.9 (C-3b), 28.3 (C-4), 14.3 (C-3c). MS (FAB, 3-NBA), *m*/*z* (%): 372.84 [M + 2] (10), 179.25 [M]^+^ (85). HRMS [M + H^+^] = calcd: 371.1044; found: 371.1054. Anal. calcd for C_19_H_15_FN_2_O_5_ (370.34): C, 61.62; H, 4.08; F, 5.13; N, 7.56. Found: C, 61.60; H, 4.06; F, 5.12; N, 7.50.

###### 4.1.2.2.8 Ethyl 2-amino-9-bromo-4-(furan-2-yl)-5-oxo-5,6-dihydro-4*H*-pyrano[3,2-*c*]quinoline-3-carboxylate (3h)

Compound 3h was obtained as brown crystals, (CHCl_3_/EtOH); yield: 85%; m.p. 320–322 °C. IR (KBr): *ν*_max_/cm^−1^ = 3462–3465 (NH_2_), 3330 (NH), 3000 (CH-Ar), 2965 (CH-Aliph), 1690 (C=O, ester), 1652 (C=O, quinolone), 1574 (C=C), 1433 (CH_2_), 1370 (CH_3_). ^1^H NMR (DMSO-*d*_6_, ppm): *δ*_H_ = 11.93 (s, 1H, NH), 7.98 (s, 1H, H-10), 7.81 (b, 2H, NH_2_), 7.37 (d, *J* = 0.8 Hz, 1H, H-5′), 7.56 (d, *J* = 8.2 Hz, 1H, H-7), 7.42 (d, *J* = 8.3 Hz, 1H, H-8), 6.18 (d, *J* = 1.8 Hz, 1H, H-3′), 6.03 (d, *J* = 2.0, 1.0 Hz, 1H, H-4′), 5.01 (s, 1H, H-4), 4.08 (q, *J* = 7.1 Hz, 2H, H-3b), 1.16 (t, *J* = 7.1 Hz, 3H, H-3c). ^13^C NMR (DMSO-*d*_6_, ppm): *δ*_C_ = 167.3 (C-3a), 160.5, 160.5 (C-2, C-2′), 158.4 (C-5), 155.0 (C-10b), 142.8 (C-5′), 138.2 (C-6a), 132.2 (C-8), 126.7 (C-10), 121.6, 121.5 (C-7, C-10a), 115.2, 110.7, 106.7 (C-9, C-4′, C-3′), 100.5 (C-4a), 75.5 (C-3), 61.3 (C-3b), 28.5 (C-4), 14.1 (C-3c). MS (FAB, 3-NBA), *m*/*z* (%): 431.64 [M]^+^ (30), 149.78 [M]^+^ (100). Anal. calcd for C_19_H_15_BrN_2_O_5_ (431.24): C, 52.92; H, 3.51; Br, 18.53; N, 6.50. Found: C, 53.02; H, 3.48; Br, 18.55; N, 6.40.

### 4.2. Biological screening

#### 4.2.1. Cytotoxic activity

The anticancer effect of the various chemical compounds was assayed using the human lung adenocarcinoma cell line A549. Cells were obtained from ATCC, cultured and maintained in DMEM high glucose (Biowest, France) supplemented with 10% FBS (Biowest, France), 100 U per ml penicillin and 1% streptomycin. Cancer cells were seeded at a density of 5 × 10^3^ cells per well in sterile 96 well flat bottom tissue culture plates the day before treatment. After allowing the cells to adhere for 24 h, serial dilutions of the tested compounds were added to the seeded cells, and the plates were incubated for 72 h at 37 °C in a 5% CO_2_ incubator. After incubation, MTT (Biobasic, Canada) dissolved in PBS was added to each well at a final concentration of 0.5 mg ml^−1^, and the plates were then incubated at 37 °C for 3 h in the dark. The MTT solution was then removed, 100 μl DMSO was added to dissolve the formed formazan crystals, and the absorbance was measured using a microplate reader (BioTek, USA). The percentage viability was calculated for each concentration relative to the control DMSO-treated cells. Graphpad software was used to calculate the IC_50_ values of each of the tested compounds.

#### 4.2.2. Topoisomerase II inhibition

The topoisomerase II enzyme inhibitory activity was performed using the *in vitro* toxicology kit assay, MTT based, Stock No. TOX-1 (catalog no. M-5655) (Sigma ®), according to the manufacturer's instructions.

#### 4.2.3. Antibacterial activity

To determine MIC, the broth microdilution method was employed through Mueller-Hinton broth, as outlined in the approved standard. The compounds showing the highest activity (as found by applying the agar diffusion method) were two-fold serially diluted in a round bottom 96 well plate to obtain a range of final concentration in the well from 1250 μg ml^−1^ to 1.22 mg ml^−1^. For bacterial suspension preparation, the standardized inoculum was prepared from a fresh plate of ATCC 6538 (the standard *Staphylococcus aureus* strain). The direct colony suspension method was used, as described in CLSI.^35^ Briefly, the bacterial suspension was prepared in 0.9% normal saline and adjusted visually to obtain turbidity equivalent to a 0.5 McFarland standard (1–2 × 10^8^ (CFU) mL^−1^). The obtained bacterial inoculum was further diluted 1 : 100 in double strength cation adjusted Mueller–Hinton broth to reach 1-2x 10^6^ CFU ml^−1^. After that, 100 ml of the prepared bacterial suspension was transferred with equal volume to the serially diluted compounds (100 μl) present in the 96 well plates. This leads to a final bacterial density in the wells of 5–10 × 10^5^ CFU ml^−1^. Additional wells for broth growth positive and negative controls containing either only bacterial suspension or uninoculated broth were included in each test. The plates were then incubated at 37 °C for 18 h. The plates were read using a microplate reader (Biotek, USA), and the MIC was determined to be the lowest concentration that inhibited bacterial growth. For solvent control, the MIC of the used DMSO was determined using the same experimental settings, as described above. Each experiment was performed at least twice.

*CLSI. Methods for Dilution Antimicrobial Susceptibility Tests for Bacteria That Grow Aerobically; Approved Standard—Eleventh Edition. CLSI document M07. Wayne, PA: Clinical and Laboratory Standards Institute. 2018.*

#### 4.2.4. Bacterial DNA gyrase inhibition

*In vitro* VEGFR-2 kinase inhibitory activity was evaluated using purified *E. coli* DNA Gyrase and Relaxed DNA Kit, Protocol TG2000GKIT, (TopoGEN, Inc.® Florida 32128, USA), following the manufacturer's instructions.

#### 4.2.5. Apoptotic investigation and cell cycle analysis

Lung epithelial cancer cells A549 were seeded into six-well culture plates (3–5 × 105 cells per well) and incubated overnight. The cells were then treated with the target compounds for 48 h. Next, media supernatants and cells were collected and rinsed with ice-cold PBS. The next step was suspending the cells in 100 μl of annexin binding buffer solution “25 mM CaCl_2_, 1.4 M NaCl, 0.1 M Hepes/NaOH, and pH 7.4” and incubation |5495471INTRNODU with “Annexin V- FITC solution (1 : 100) and propidium iodide (PI)” at a concentration of 10 μg ml^−1^ in the dark for 30 min. The stained cells were then acquired by applying a Cytoflex FACS machine. Data were analyzed using cytExpert software.^36^

### 4.3. Molecular docking

The docking scores and binding modes of the newly synthesized compounds with the target proteins (DNA gyrase and topoisomerase II) were predicted by employing the MOE 2019.10259 software.

### 4.4. Investigated derivative preparation

First, Chemdraw software was employed to draw the tested compounds, which were then imported to an optimized database, as previously discussed.^37^

### 4.5. Protein preparation

3D crystal structures of the target proteins (PDB: 5GWK^31^ and PDB: 2XCT^32^) were obtained from the protein data bank website,^38^ followed by structure preparation and optimization for established docking, as described in detail.^35^ Subsequently, docking of the assessed derivatives 3a–h to the target protein complex was carried out by applying the default docking protocol. For each docked structure, the pose with prominent amino acid interactions, the best docking score, and RMSD were selected and visualized.

## Data availability

Data will be made available on request. CCDC 2312660 (3a, https://www.ccdc.cam.ac.uk/structures/search?Ccdc=2312660&Author=Nieger&Access=referee) and 2312661 (3b, https://www.ccdc.cam.ac.uk/structures/search?Ccdc=2312661&Author=Nieger&Access=referee) contain the supplementary crystallographic data for this paper. These data can be obtained free of charge from The Cambridge Crystallographic Data Centre *via*www.ccdc.cam.ac.uk/data_request/cif.

## Author contributions

A. A. Aly: conceptualization, writing, and editing; H. A. Abd El-Naby, E. K. Ahmed: supervision; S. A. Gedamy: methodology, writing the draft; M. Nieger, K. Rissanen: X-ray, writing draft; Alan B. Brown: editing; Marwa M. Shaaban, Amal Atta: biology, molecular modeling, writing and editing; Michael G. Shehat: biology.

## Conflicts of interest

The authors declare no conflict of interest.

## Supplementary Material

RA-015-D4RA06201A-s001

RA-015-D4RA06201A-s002

## References

[cit1] Sharhan A. A., Ahmed Neema A.-M., Rawaa S. A.-A., Kzar H. H. (2023). South Asian Res. J. Pharm. Sci..

[cit2] Duan C., Yu M., Xu J., Li B.-Y., Zhao Y., Kankala R. K. (2023). Biomed. Pharmacother..

[cit3] Al-Jumaili M. H. A., Hamad A. A., Hashem H. E., Hussein A. D., Muhaidi M. J., Ahmed M. A., Albanaa A. H. A., Siddique F., Bakr E. A. (2023). J. Mol. Struct..

[cit4] Barta J. A., Powell C. A., Wisnivesky J. P. (2019). Ann. Glob. Health.

[cit5] Nagasaka M., Gadgeel S. M. (2018). Expert Rev. Anticancer Ther..

[cit6] Okoro C. O., Fatoki T. H. (2023). Int. J. Mol. Sci..

[cit7] Vaidya A., Jain S., Jain A. K., Prashanthakumar B., Kashaw S. K., Agrawal R. K. (2015). Med. Chem. Res..

[cit8] Baldwin E., Osheroff N. (2005). Curr. Med. Chem.: Anti-Cancer Agents.

[cit9] Upadhyay K. D., Dodia N. M., Khunt R. C., Chaniara R. S., Shah A. K. (2018). ACS Med. Chem. Lett..

[cit10] Almalki F. A. (2023). Saudi Pharm. J..

[cit11] Fu H.-G., Li Z.-W., Hu X.-X., Si S.-Y., You X.-F., Tang S., Wang Y.-X., Song D.-Q. (2019). Molecules.

[cit12] Ramadan M., Abd El-Aziz M., Elshaier Y. A., Youssif B. G., Brown A. B., Fathy H. M., Aly A. A. (2020). Bioorg. Chem..

[cit13] Pradhan V., Kumar R., Mazumder A., Abdullah M. M., Shahar Yar M., Ahsan M. J., Ullah Z. (2023). Chem. Biol. Drug Des..

[cit14] Grossman S., Fishwick C. W., McPhillie M. J. (2023). Pharmaceuticals.

[cit15] Wei Y., Sandhu E., Yang X., Yang J., Ren Y., Gao X. (2022). Microorganisms.

[cit16] Koslow M., Shochet G. E., Matveychuk A., Israeli-Shani L., Guber A., Shitrit D. (2017). J. Thorac. Dis..

[cit17] Gotland N., Uhre M. L., Sandholdt H., Mejer N., Lundbo L. F., Petersen A., Larsen A. R., Benfield T. (2020). Medicine.

[cit18] Hattar K., Reinert C. P., Sibelius U., Gökyildirim M. Y., Subtil F. S. B., Wilhelm J., Eul B., Dahlem G., Grimminger F., Seeger W., Grandel U. (2017). Cancer Immunol., Immunother..

[cit19] Qi J. L., He J. R., Liu C. B., Jin S. M., Gao R. Y., Yang X., Bai H. M., Ma Y. B. (2020). MedComm.

[cit20] Asghari S., Ramezani S., Mohseni M. (2014). Chin. Chem. Lett..

[cit21] Jayagobi M., Raghunathan R., Sainath S., Raghunathan M. (2011). Eur. J. Med. Chem..

[cit22] Mathada B. S. D., Mathada M. B. H. (2009). Chem. Pharm. Bull..

[cit23] Watpade R., Bholay A., Toche R. (2017). J. Heterocycl. Chem..

[cit24] Pham T. D. M., Ziora Z. M., Blaskovich M. A. T. (2019). MedChemComm.

[cit25] Abd El-Lateef H. M., Elmaaty A. A., Abdel Ghany L. M., Abdel-Aziz M. S., Zaki I., Ryad N. (2023). ACS Omega.

[cit26] Elbastawesy M. A. I., Mohamed F. A. M., Zaki I., Alahmdi M. I., Alzahrani S. S., Alzahrani H. A., Gomaa H. A. M., Youssif B. G. M. (2023). J. Mol. Struct..

[cit27] Spencer A. C., Panda S. S. (2023). Biomedicines.

[cit28] Khan T., Sankhe K., Suvarna V., Sherje A., Patel K., Dravyakar B. (2018). Biomed. Pharmacother..

[cit29] Meyler's Side Effects of Drugs, ed. J. K. Aronson, Elsevier, Oxford, 16th edn, 2016, pp. 470–471, 10.1016/B978-0-444-53717-1.01465-7

[cit30] Oliveira L., Langoni H., Hulland C., Ruegg P. (2012). J. Dairy Sci..

[cit31] Wang Y. R., Chen S. F., Wu C. C., Liao Y. W., Lin T. S., Liu K. T., Chen Y. S., Li T. K., Chien T. C., Chan N. L. (2017). Nucleic Acids Res..

[cit32] Bax B. D., Chan P. F., Eggleston D. S., Fosberry A., Gentry D. R., Gorrec F., Giordano I., Hann M. M., Hennessy A., Hibbs M., Huang J., Jones E., Jones J., Brown K. K., Lewis C. J., May E. W., Saunders M. R., Singh O., Spitzfaden C. E., Shen C., Shillings A., Theobald A. J., Wohlkonig A., Pearson N. D., Gwynn M. N. (2010). Nature.

[cit33] Bhudevi B., Ramana P. V., Mudiraj M., Reddy A. R. (2009). Indian J. Chem., Sect. B.

[cit34] Mohamed E. A., Ismail M. M., Gabr Y., Abass M. (1994). Chem. Pap..

[cit35] Chandrasekaran S., Abbott A., Campeau S., Zimmer B. L., Weinstein M., Hejna J., Walker L., Kirn T., Patel R. (2018). J. Clin. Microbiol..

[cit36] Nafie M. S., Kishk S. M., Mahgoub S., Amer A. M. (2022). Chem. Biol. Drug Des..

[cit37] Al-Humaidi J. Y., Shaaban M. M., Rezki N., Aouad M. R., Zakaria M., Jaremko M., Hagar M., Elwakil B. H. (2022). Life.

[cit38] https://www.rcsb.org/

