# Supplementary material for: Microwave-assisted synthesis and in vitro and in silico studies of pyrano[3,2-c]quinoline-3-carboxylates as dual acting anti-cancer and anti-microbial agents and potential topoisomerase II and DNA-gyrase inhibitors
Source: RSC Adv. 2025 Jan 21;15(3):1941–56. doi: 10.1039/d4ra06201a (PMC11749604; doi:10.1039/d4ra06201a)
Supplement: RA-015-D4RA06201A-s001 [file RA-015-D4RA06201A-s001.pdf]

Supplementary Information (SI) for RSC Advances.
This journal is © The Royal Society of Chemistry 2024

Microwave assisted synthesis, *in vitro* and *in silico* studies of pyrano[3,2-*c*]quinoline-3-carboxylates as dual acting anti-cancer and anti-microbial agents as potential topoisomerase II and DNA-gyrase inhibitors

Ashraf A Aly,[1*] Hisham A. Abd El-Naby,[1] Essam Kh. Ahmed,[1] Sageda A. Gedamy,[1] Kari Rissanen,[2] Martin Nieger,[3] Michael G. Shehat, [4] Marwa M. Shaaban,[5] Amal Atta[5]

[1]*Chemistry Department, Faculty of Science, Minia University, 61519 El-Minia, Egypt, e-mails: A. A. Aly, ashrafaly63@yahoo.com (ashraf.shehata@mu.edu.eg); H.A. Abd El-Naby, hisham_minia@mu.edu.eg; E.K. Ahmed, essam.mohd@mu.edu.eg; S. A. Gedamy, sagedaali332@yahoo.com*

[2] *Department of Chemistry, University of Jyv\äskylä, P.O. Box 35, FIN-40014 Jyv\äskylä. Finland, K. Rissanen, e-mail: kari.t.rissanen@jyu.fi*

[3] *Department of Chemistry, University of Helsinki, P.O. Box 55, A. I. Virtasen aukio I, 00014 Helsinki, Finland, K. Rissanen, M. Nieger, e-mail: martin.nieger@helsinki.fi*

[4]*Department of Microbiology, Faculty of Pharmacy, Alexandria University, Alexandria 21521, Egypt, Michael G. Shehat, e-mail: michael.shehat@alexu.edu.eh*

[5] *Department of Pharmaceutical Chemistry, Faculty of Pharmacy, Alexandria University, Alexandria 21521, Egypt, M. M. Shaaban, e-mail: maraw.mamdouh@alexu.edu.eg; A. Atta, e-mail: amal.atta@alexu.edu.eg (missalex_pharma@yahoo.com)*

To whom correspondence should be addressed:* ***Prof Ashraf A. Aly***,** ***Ph.D****. Chemistry Department, Faculty of Science, Minia University, 61519, El-Minia, Egypt; e-mail address: ashrafaly63@yahoo.com (ashraf.shehata@mu.edu.eg).*

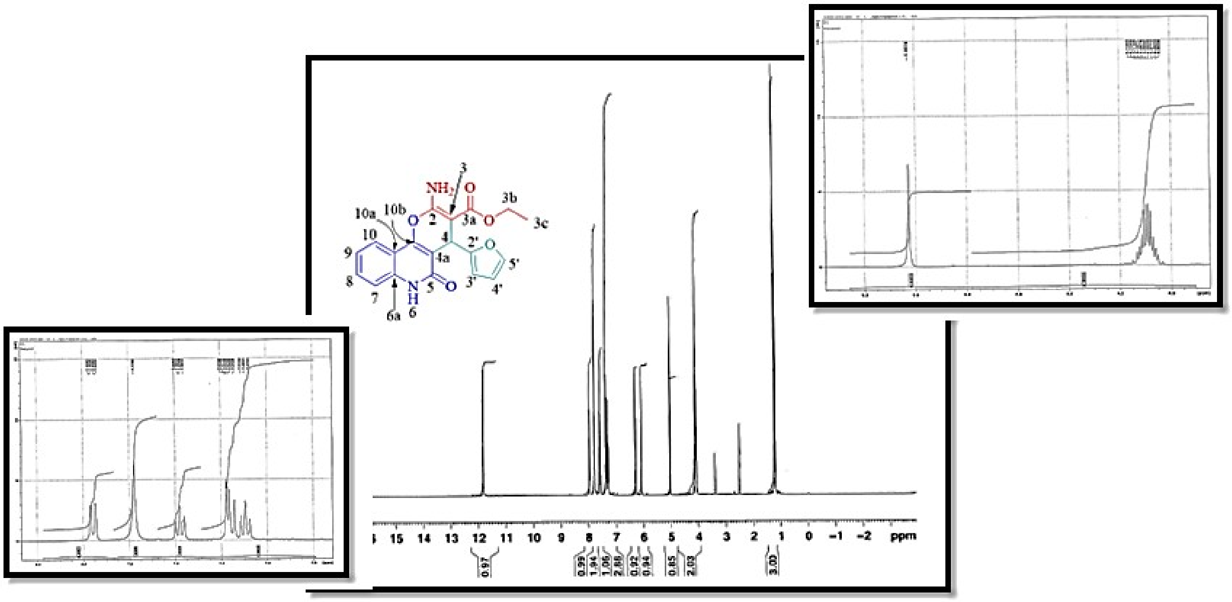

SI Figure 1. ^{1}H NMR spectrum of compound **3a**

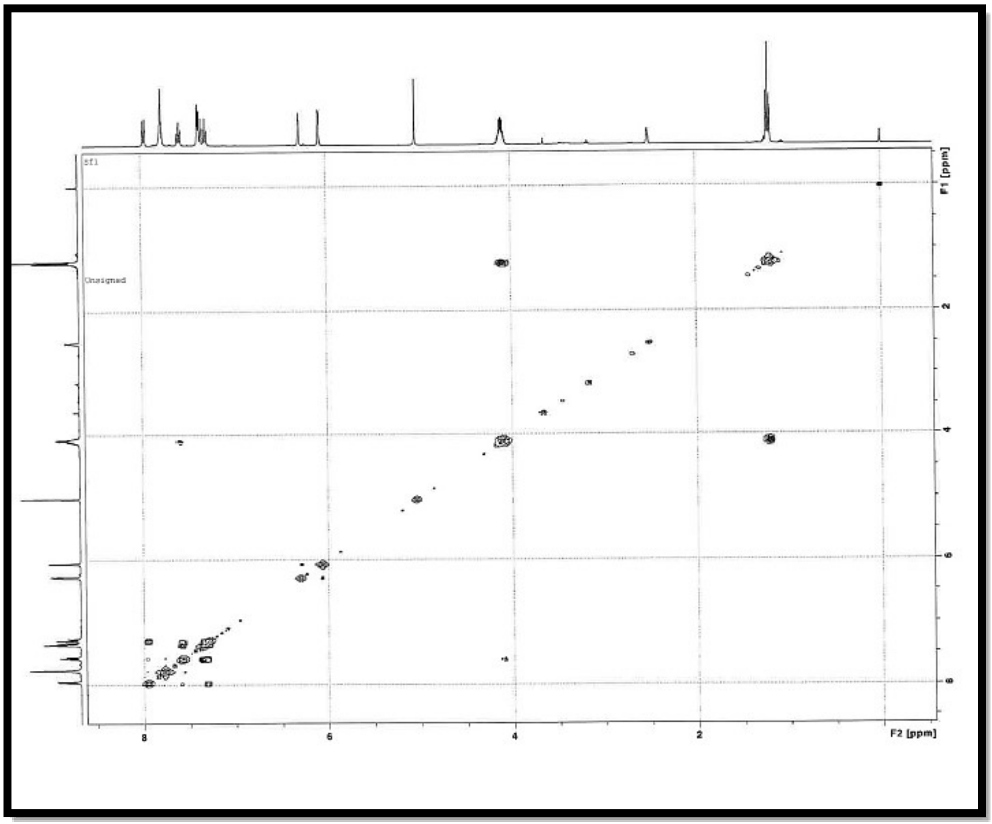

SI Figure 2. ^{1}H -^{1}H- COSY NMR of compound **3a**

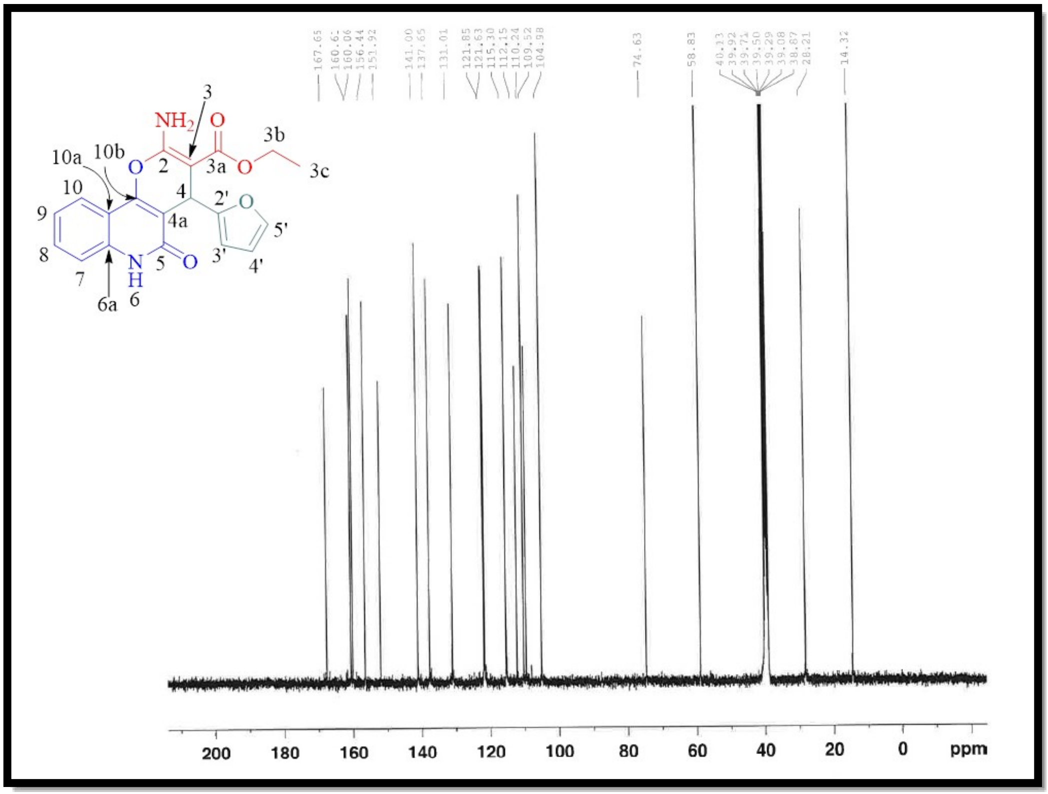

SI Figure 3. ^{13}C NMR spectrum of compound **3a**

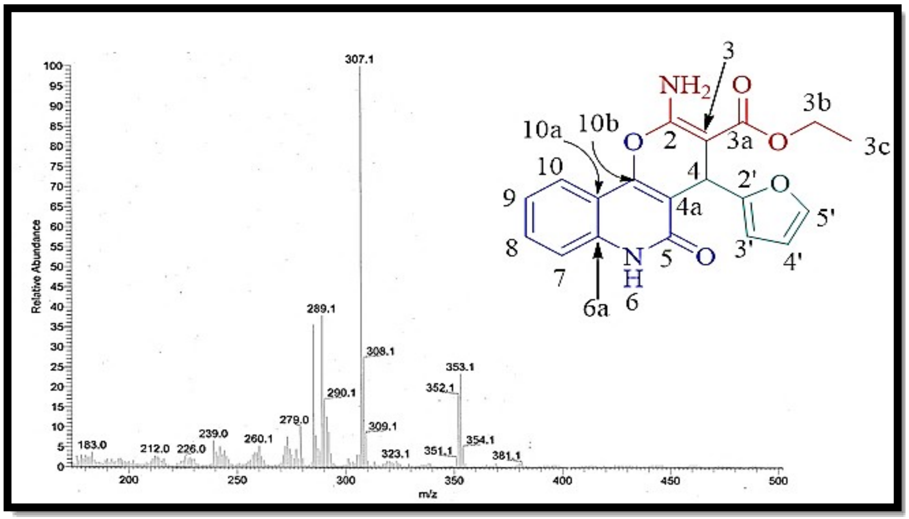

SI Figure 4. Mass spectroscopy of compound **3a**

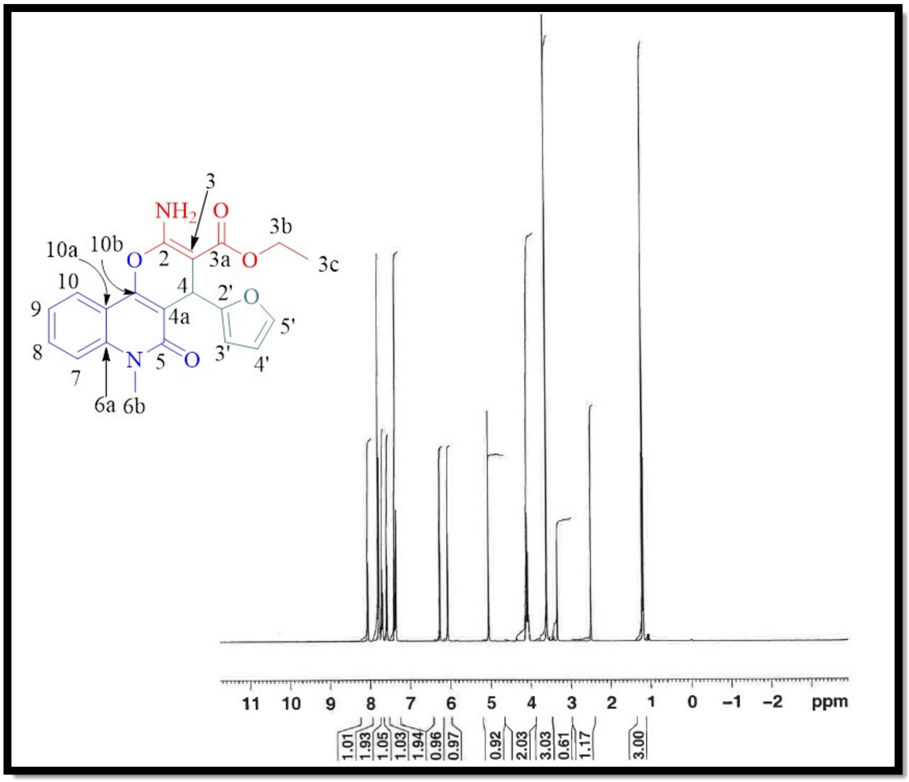

SI Figure 5. ^{1}**H** NMR spectrum of compound **3b**

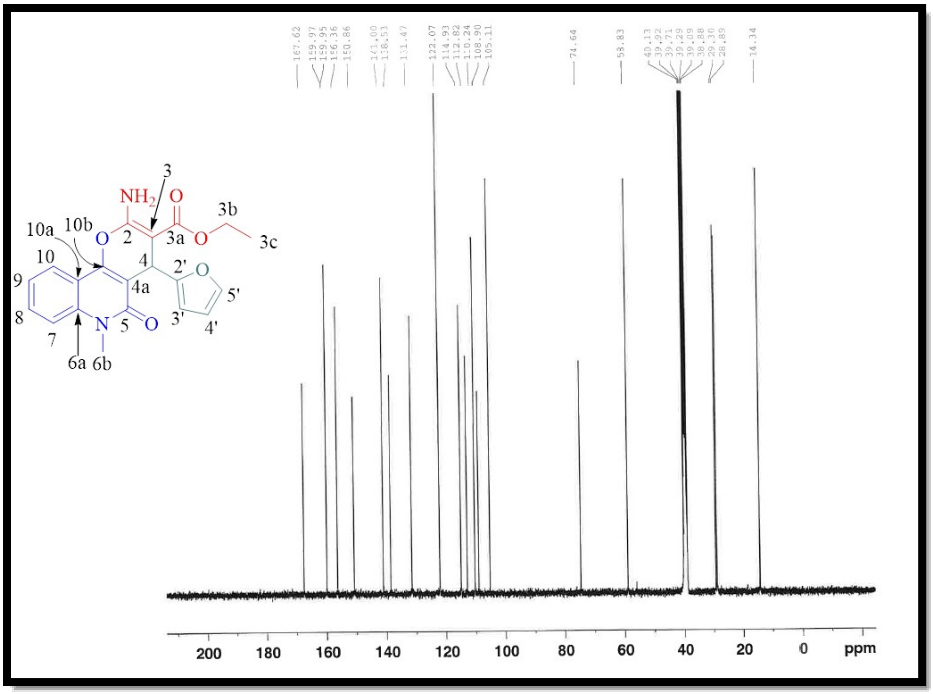

SI Figure 6. ^{13}C NMR spectrum of compound **3b**

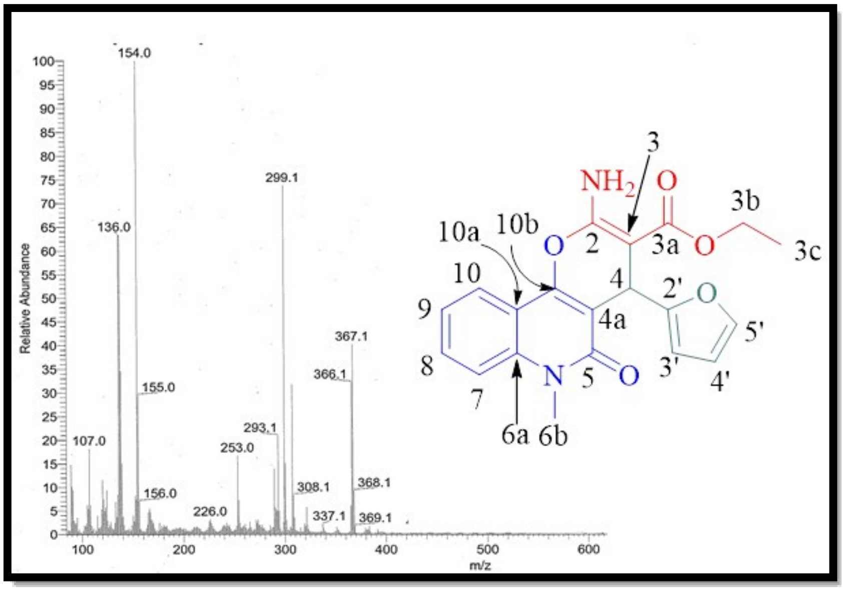

SI Figure 7. Mass spectrum of compound **3b**

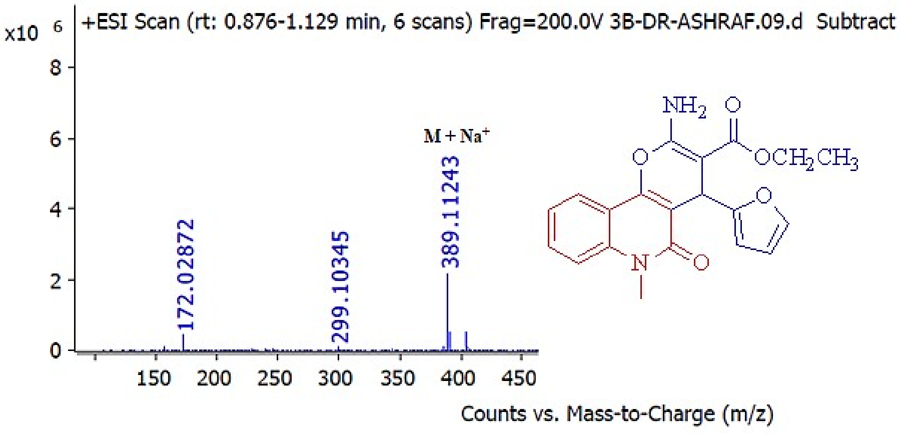

SI Figure 8. HRMS spectroscopy of compound **3b**

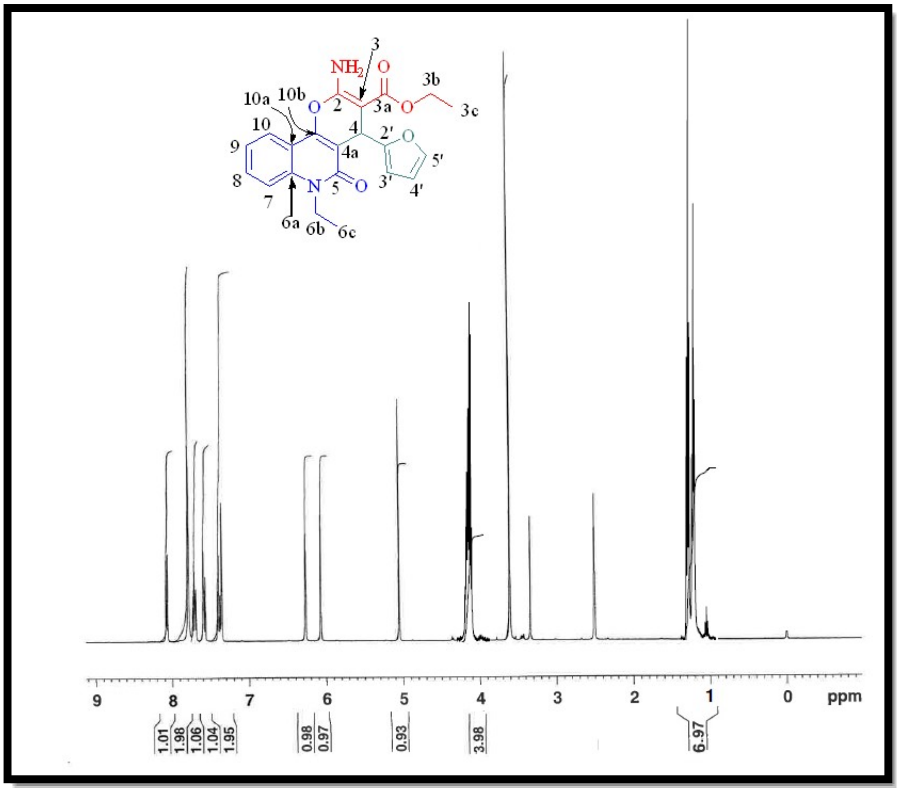

SI Figure 9. **^{1}H** NMR spectrum of compound **3c**

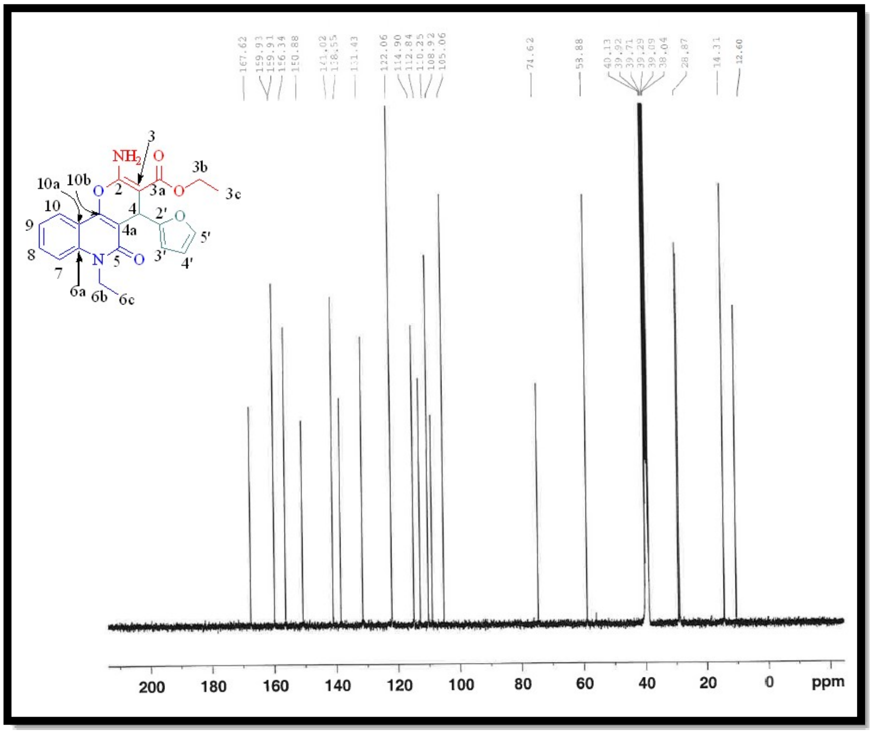

SI Figure 10. ^{13}C NMR spectrum of compound **3c**

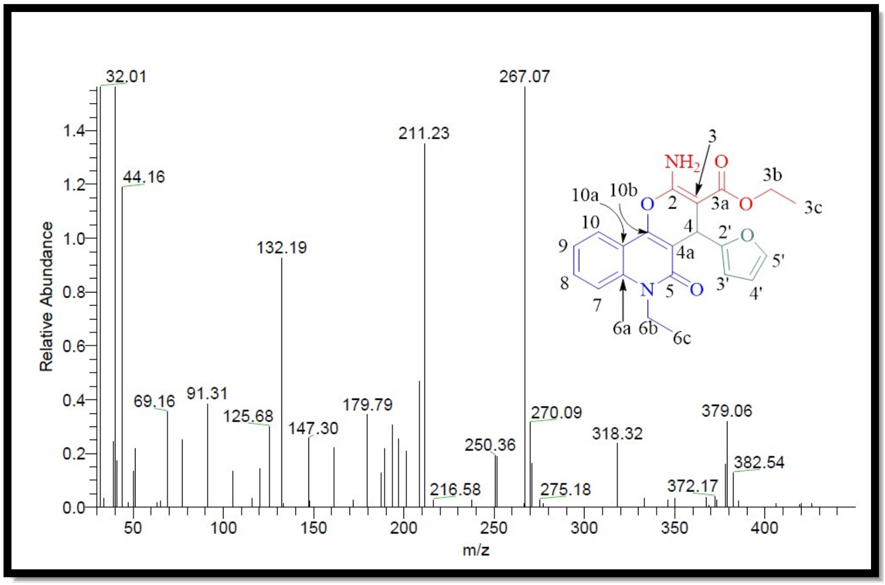

SI Figure 11. Mass spectrum of compound **3c**

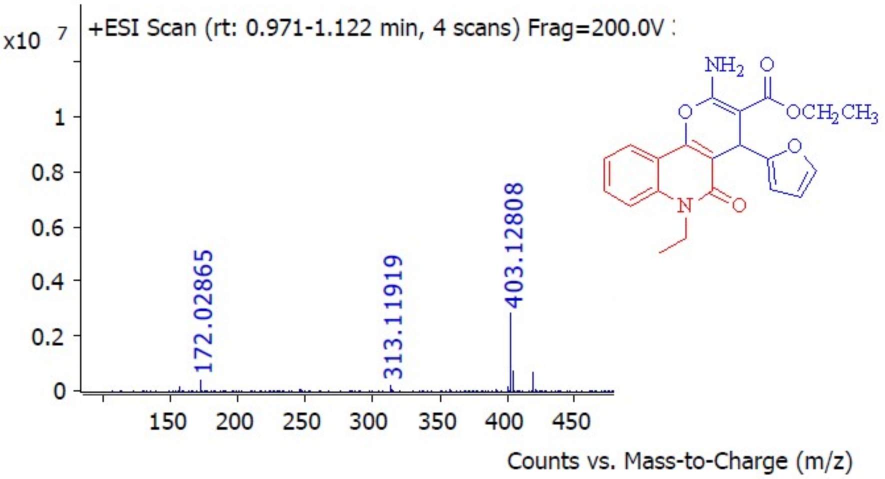

SI Figure 12. HRMS of spectroscopy of compound **3c**

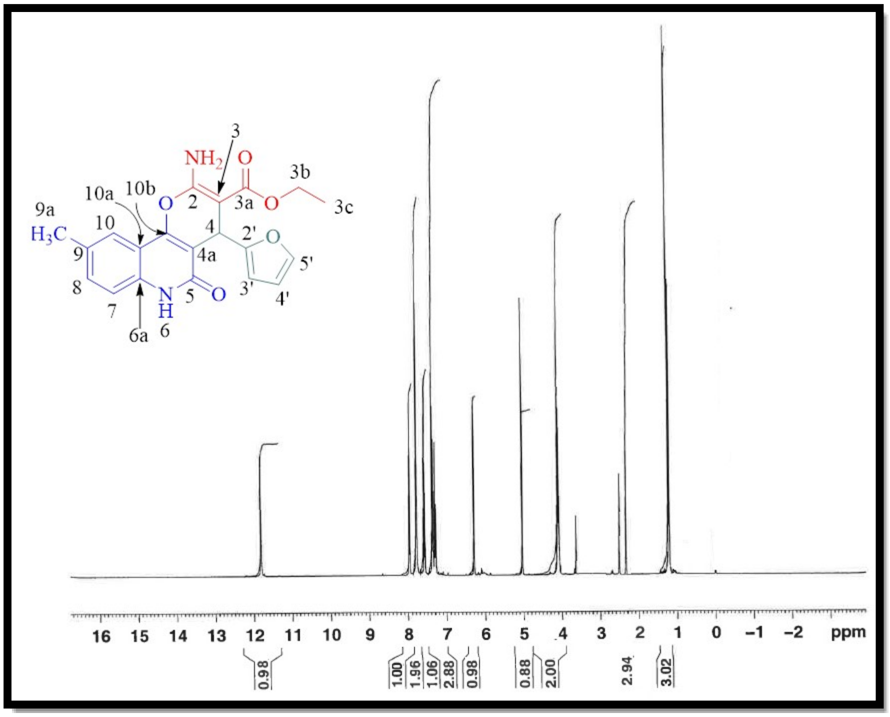

SI Figure 13. ^{1}H NMR spectrum of compound **3d**

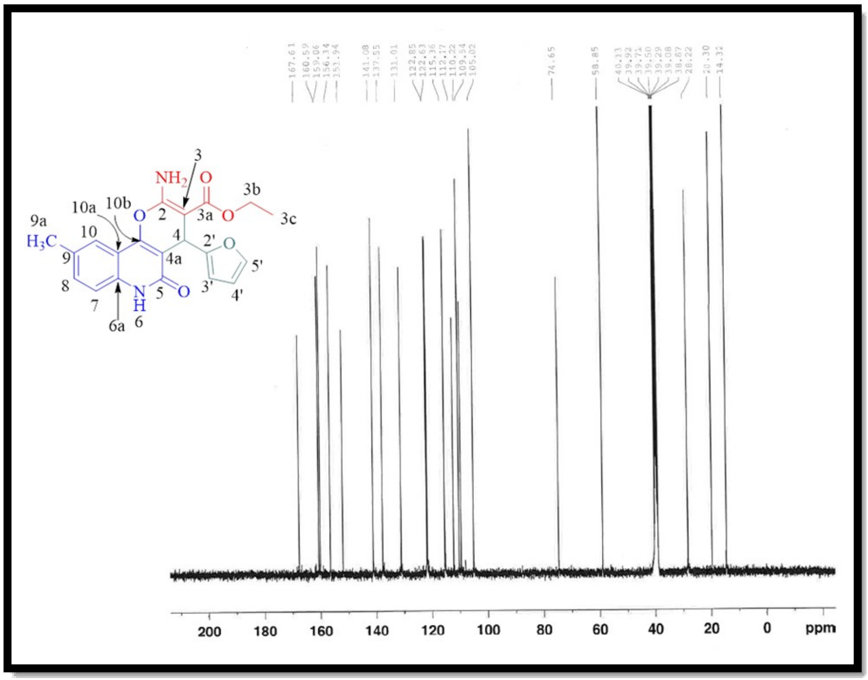

SI Figure 14. ^{13}C NMR spectrum of compound **3d**

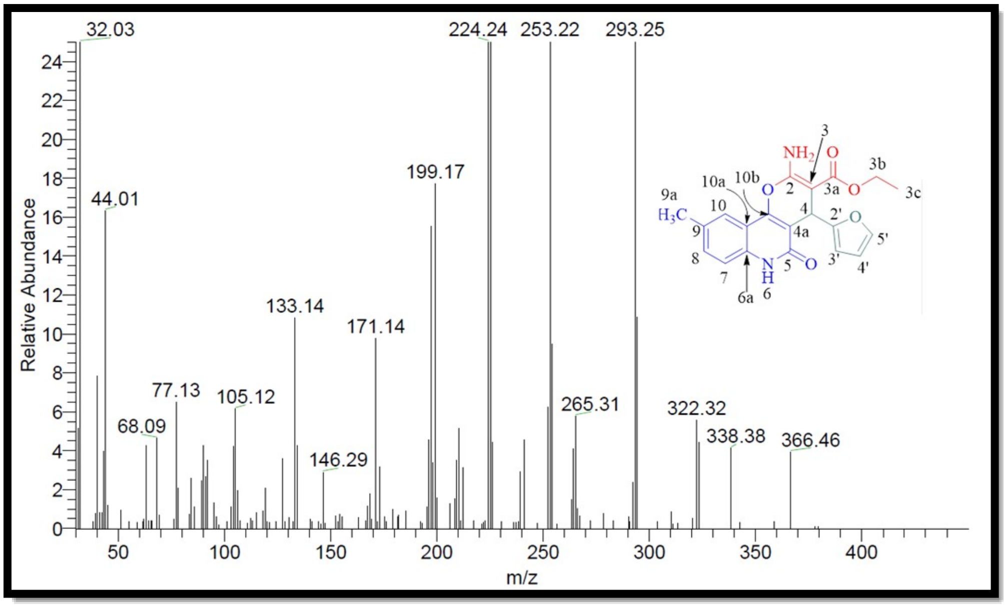

SI Figure 15. Mass spectrum of compound **3d**

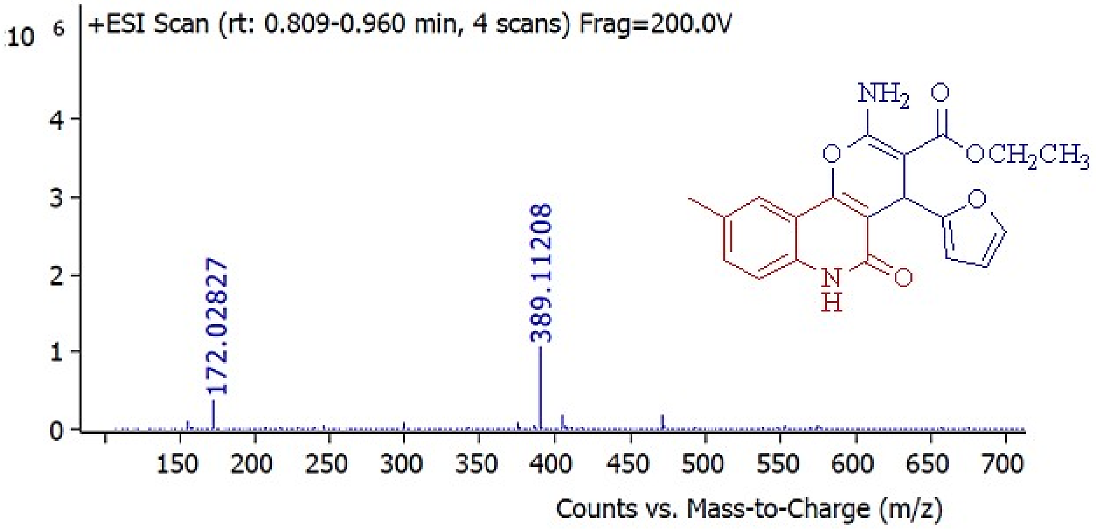

SI Figure 16. HRMS spectroscopy of compound **3d**

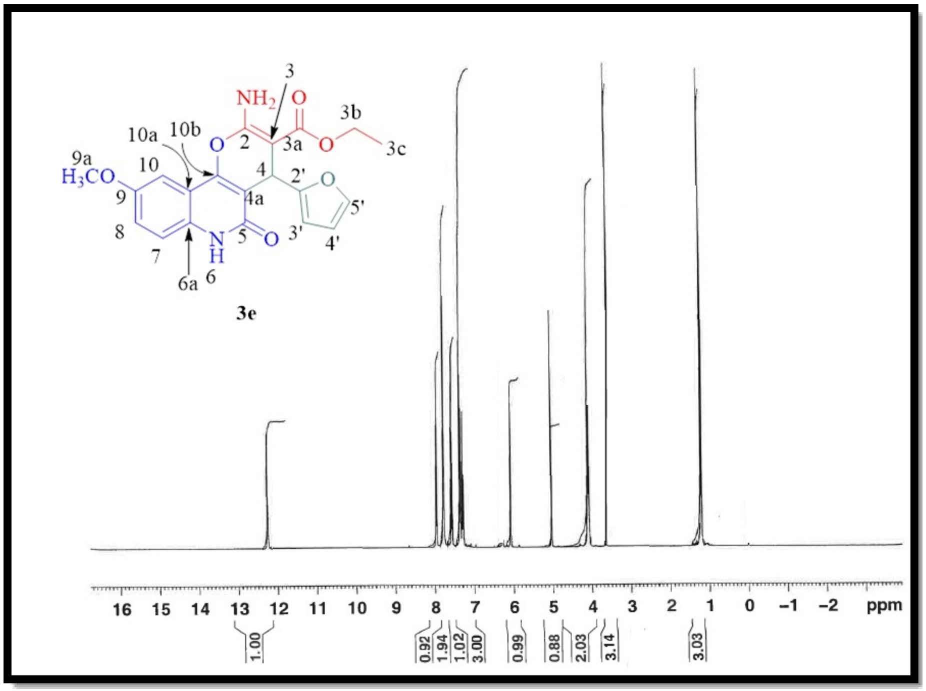

SI Figure 17. ^{1}H NMR spectrum of compound **3e**

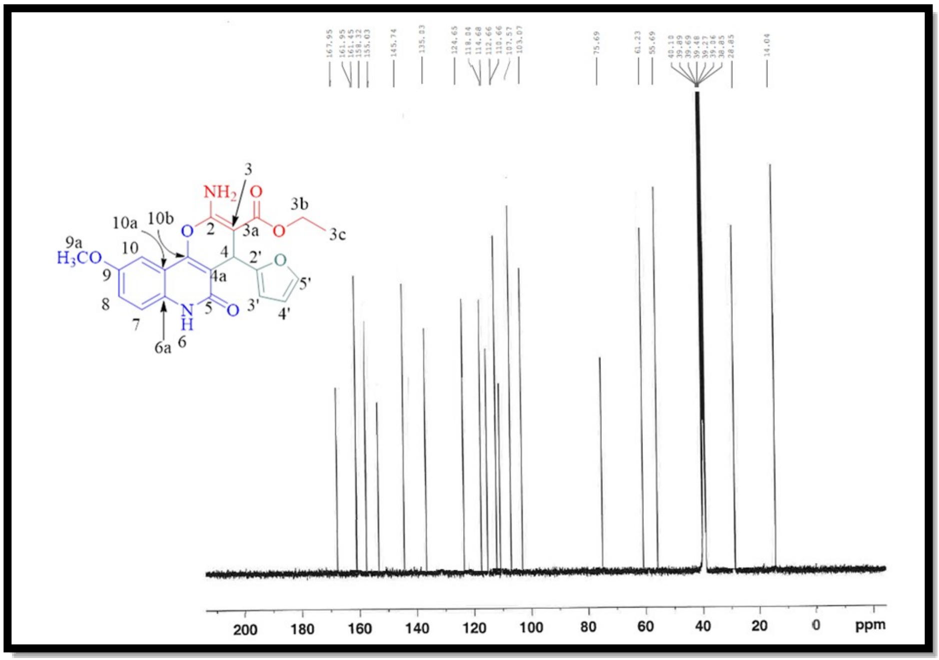

SI Figure 18. ^{13}C NMR spectrum of compound **3e**

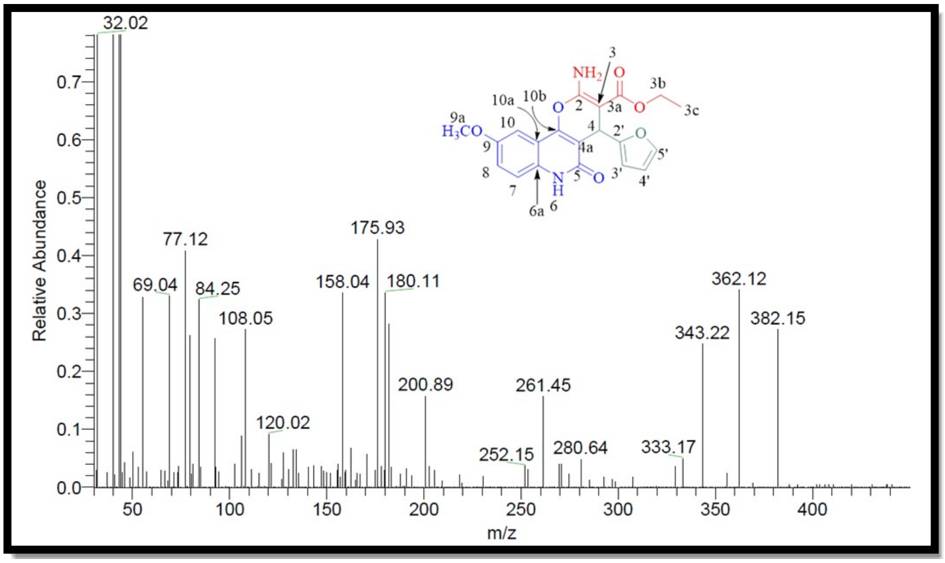

SI Figure 19. Mass spectrum of compound **3e.**

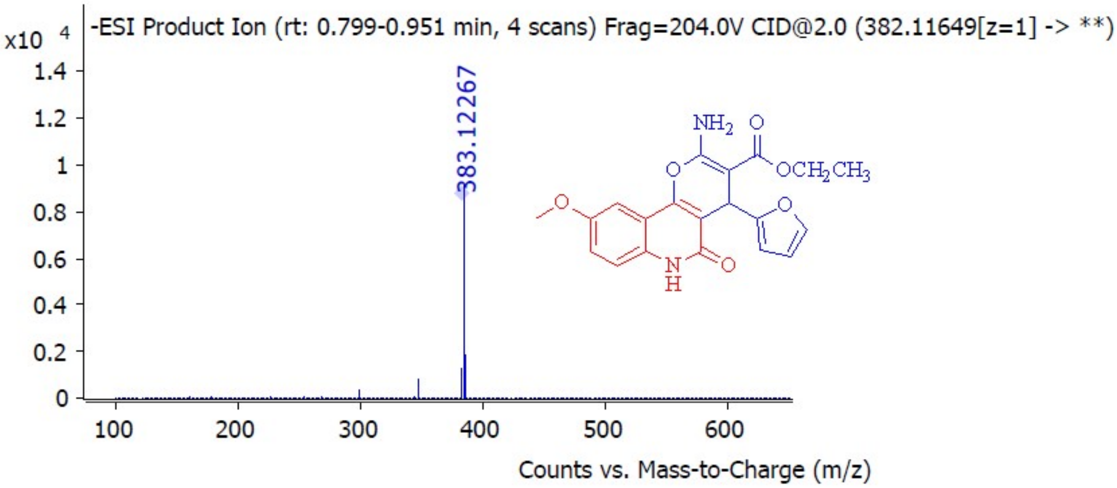

SI Figure 20. HRMS spectroscopy of compound **3e.**

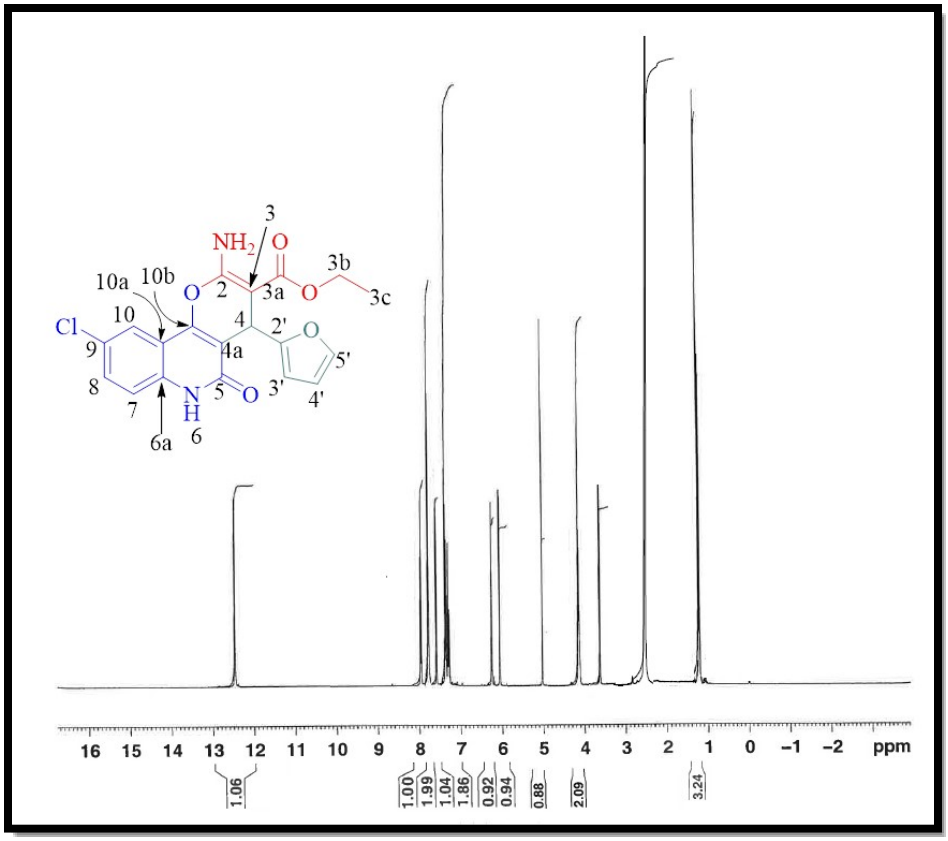

SI Figure 21. ^{1}H NMR spectrum of compound **3f**

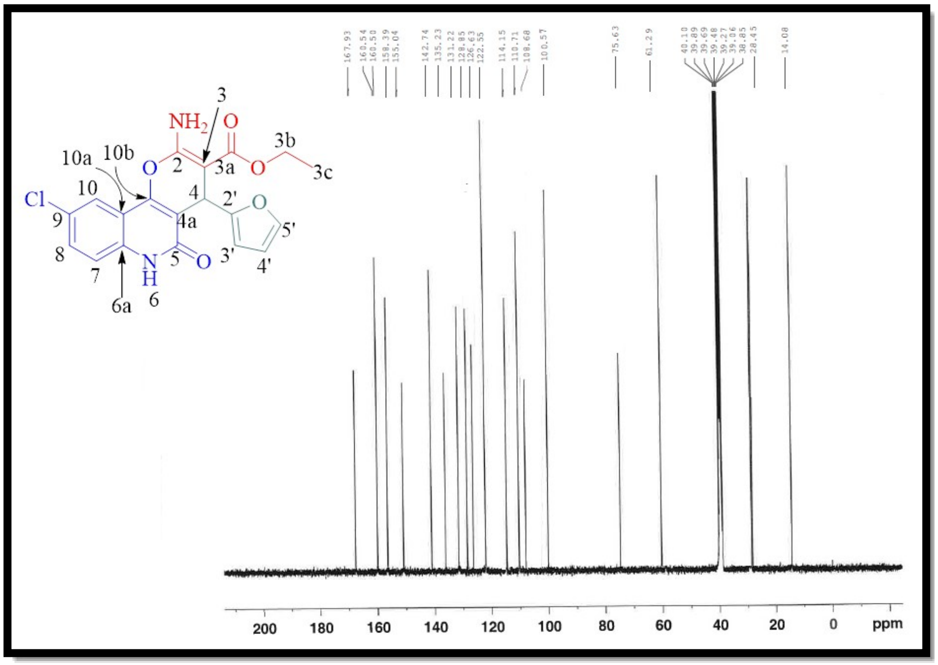

SI Figure 22. ^{13}C NMR spectrum of compound **3f**

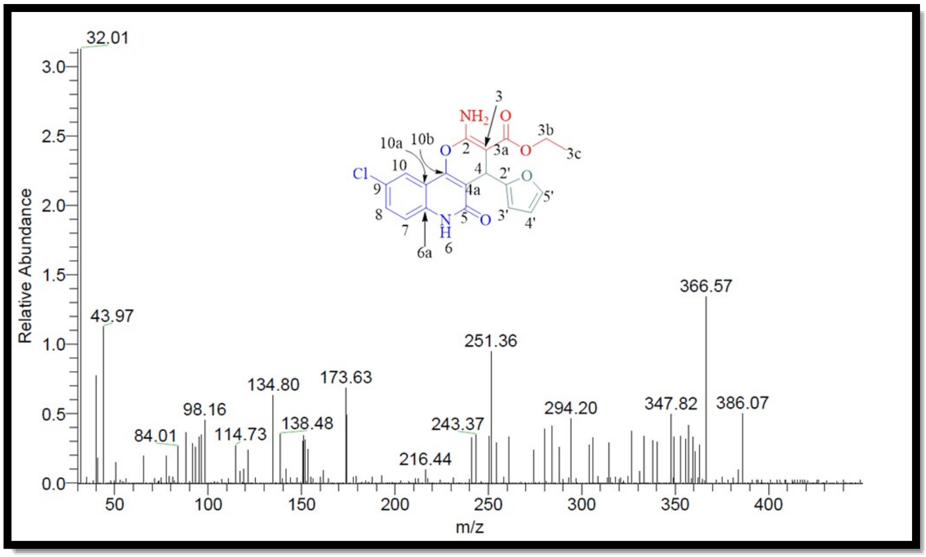

SI Figure 23. Mass spectrum of compound **3f**

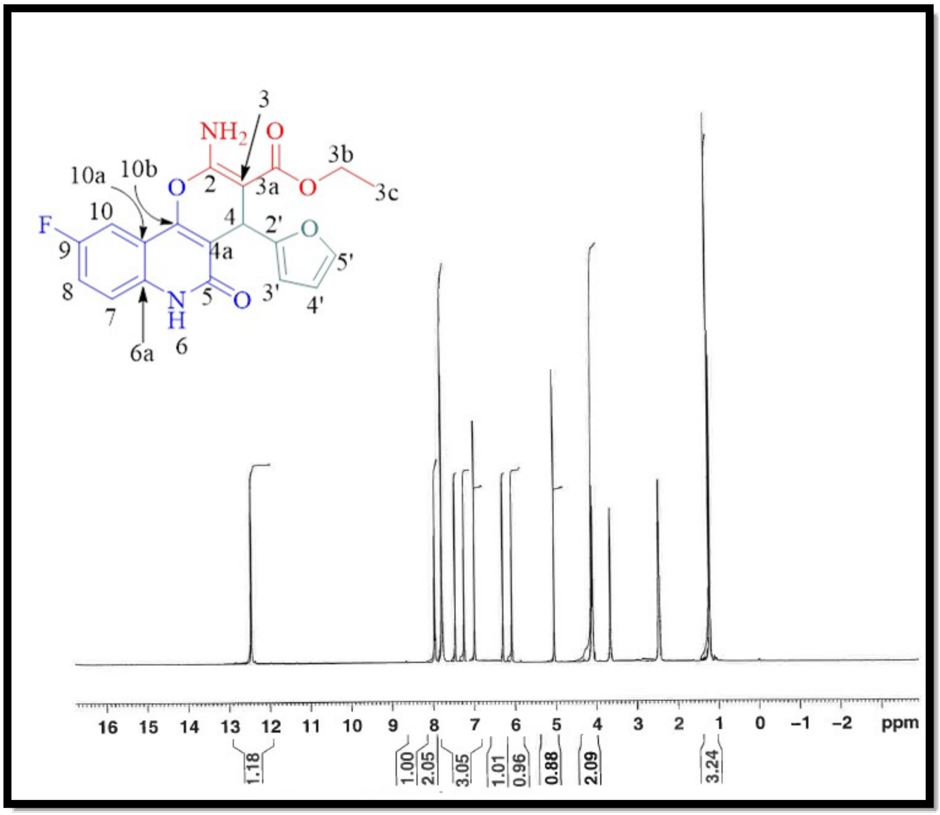

SI Figure 24. ^{1}H NMR spectrum of compound **3g**

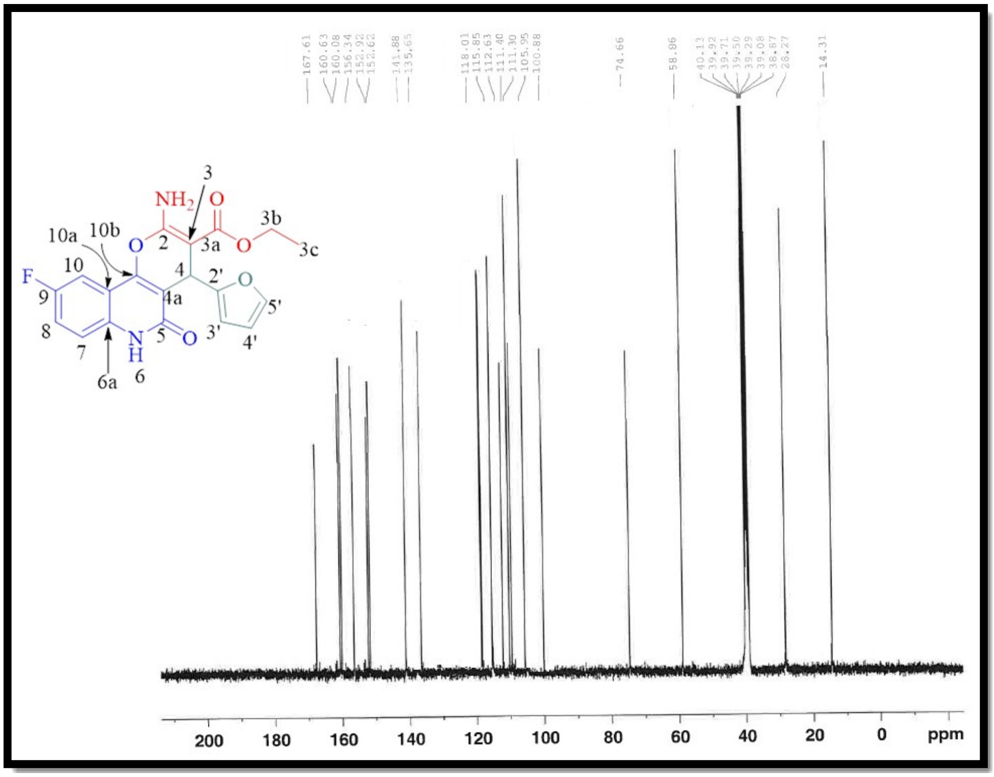

SI Figure 25. ^{13}C NMR spectrum of compound **3g**

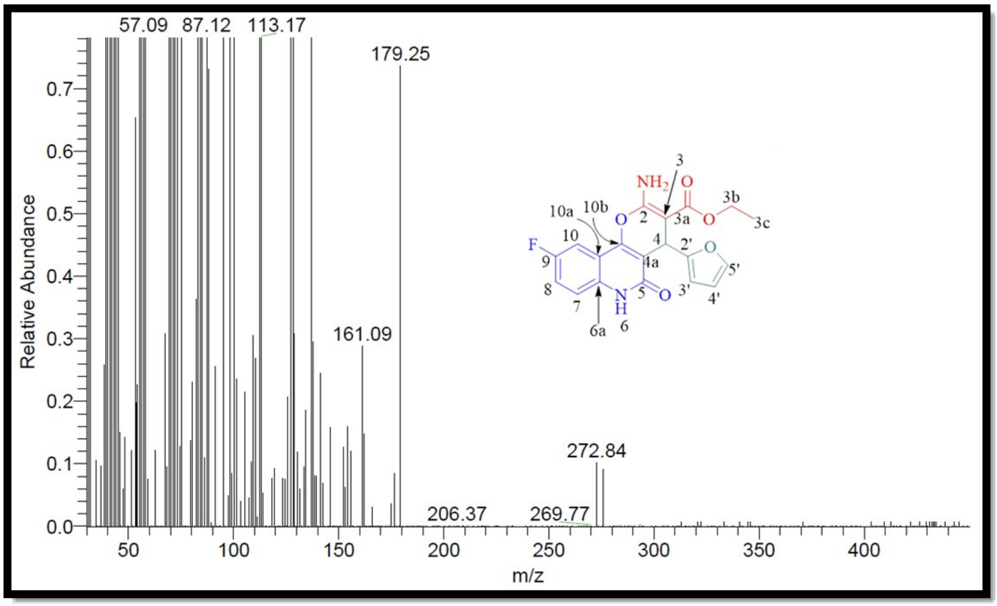

SI Figure 26. Mass spectrum of compound **3g**

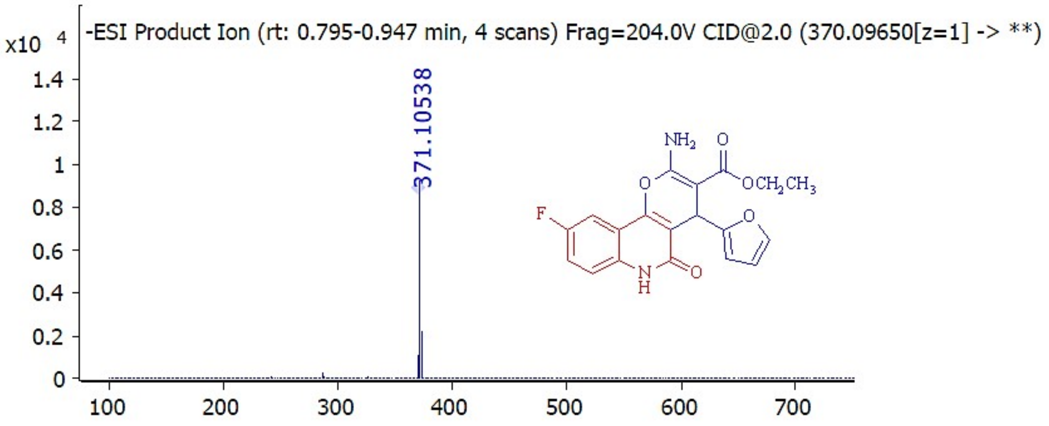

SI Figure 27. HRMS spectroscopy of compound **3g**

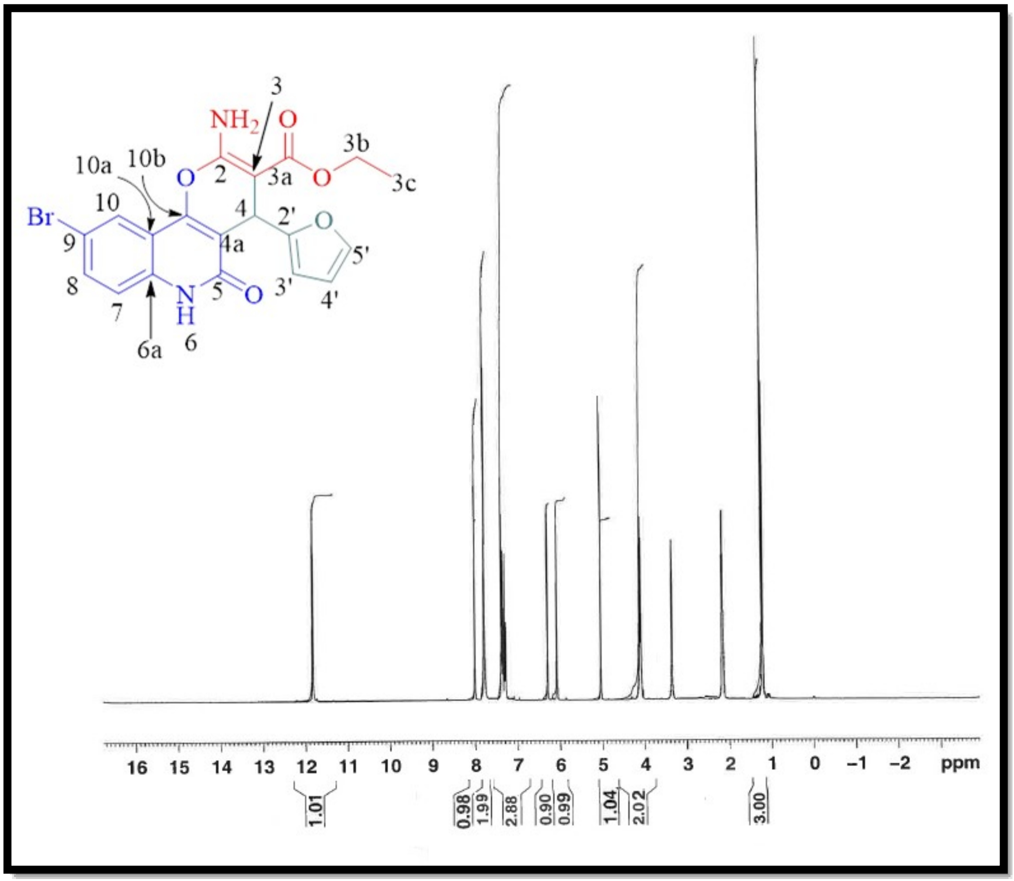

SI Figure 28. ^{1}H NMR spectrum of compound **3h**

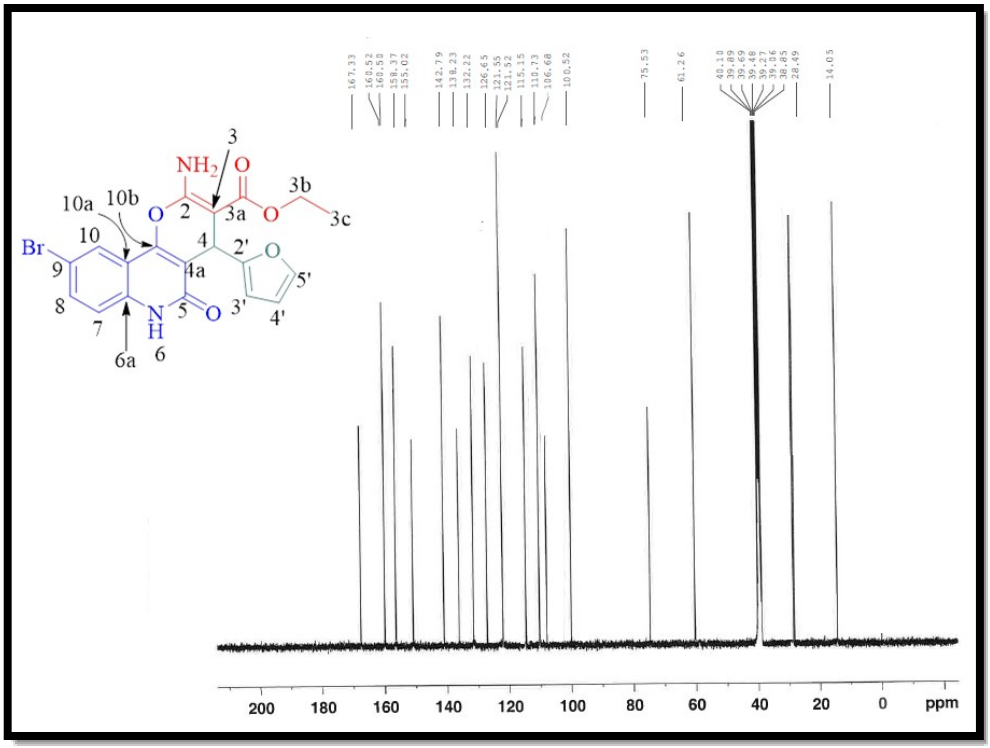

SI Figure 29. ^{13}C NMR spectrum of compound **3h**

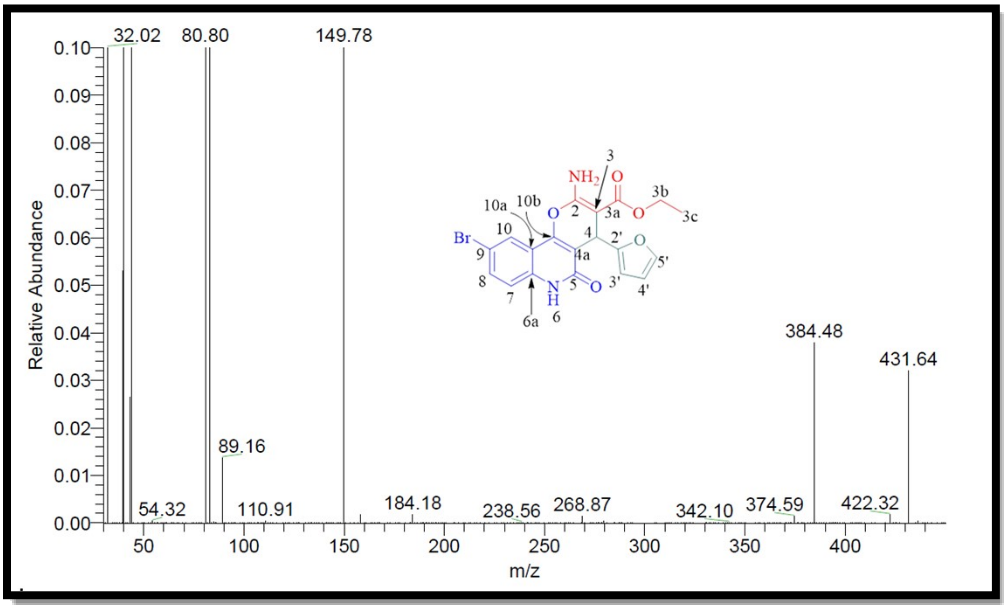

SI Figure 30. Mass spectrum of compound **3h**

Docking study

Compound	Topo II	Binding interactions with Topo II	DNA gyrase	Binding interactions with DNA Gyrase
**3a**	-7.63	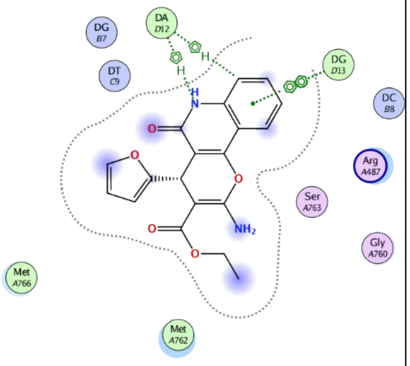	-8.14	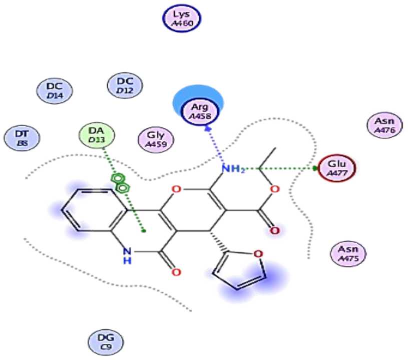
**3b**	-7.72	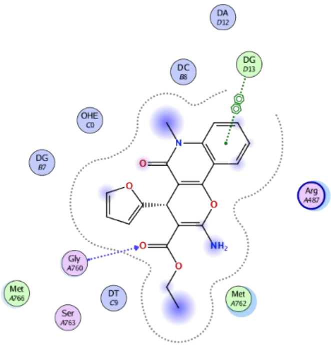	-8.27	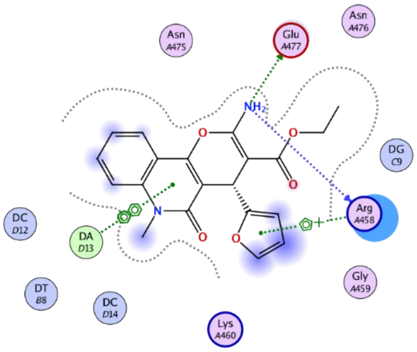
**3c**	-8.01	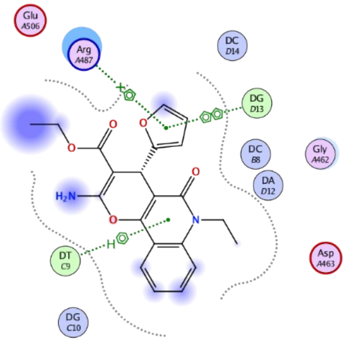	-8.26	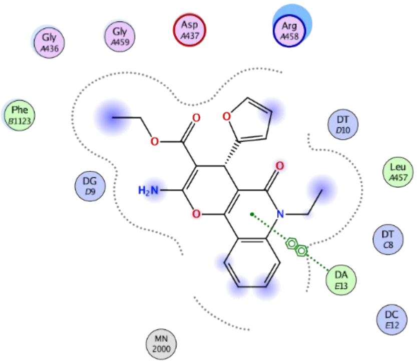

**3d**	-7.90	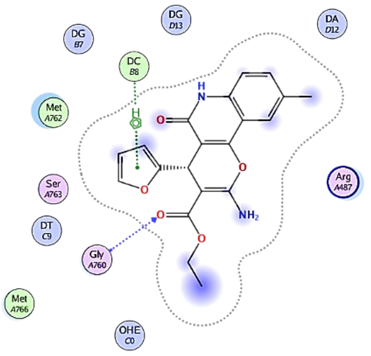	-8.55	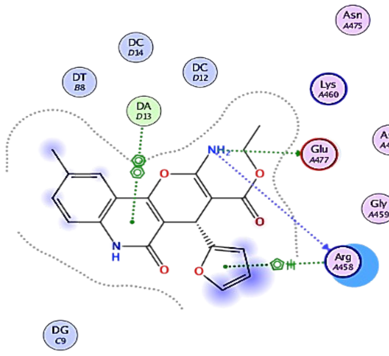
**3e**	-8.19	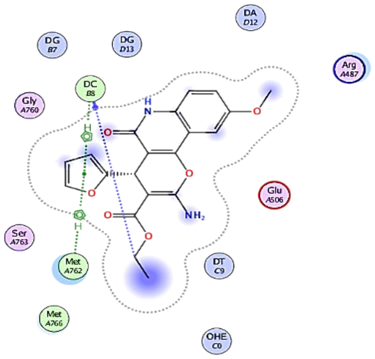	-8.31	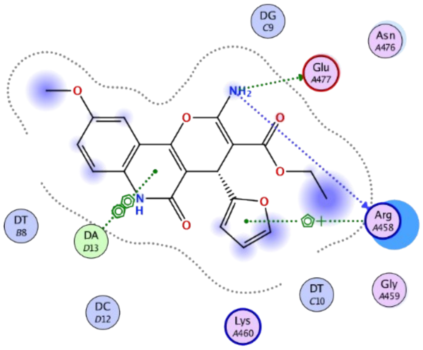
**3f**	-7.96	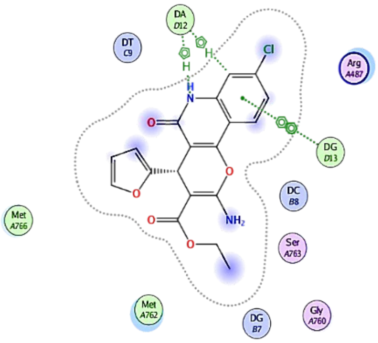	-8.45	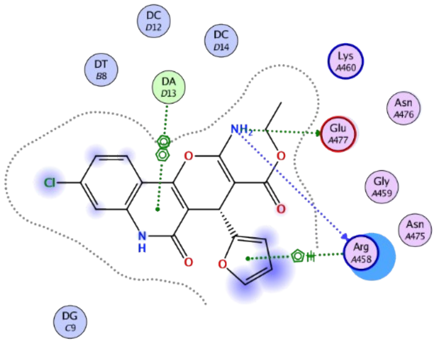
**3g**	-7.55	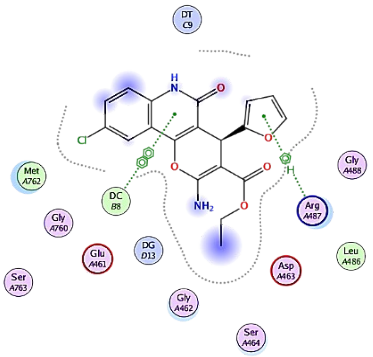	-8.25	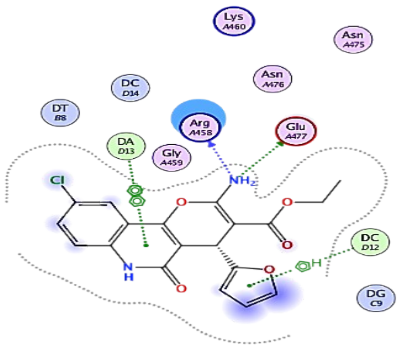

**3h**	-7.80	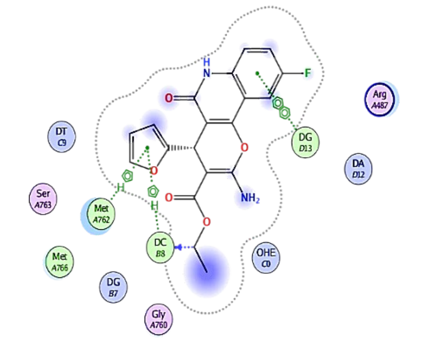	-8.03	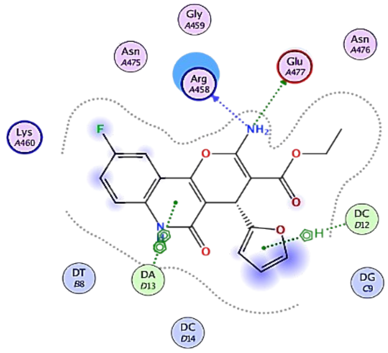
**3i**	**-7.82**	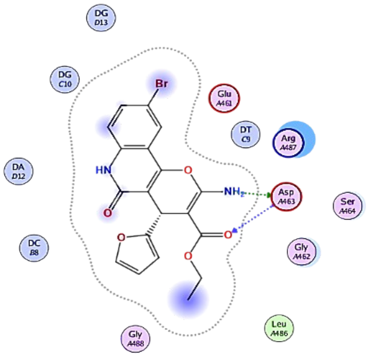	**-8.63**	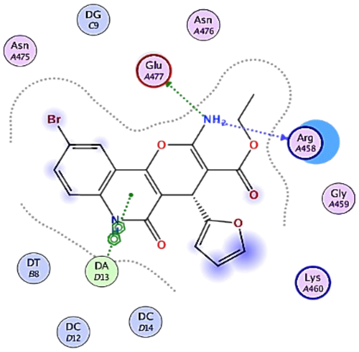
**Co-crystallized ligand**	**-11.02 (0.35)**	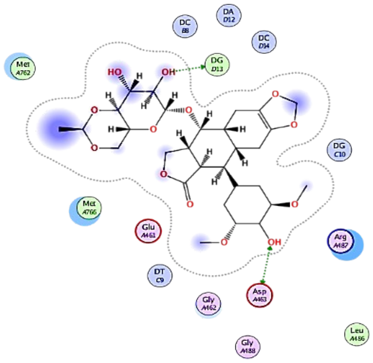	**-9.92 (0.64)**	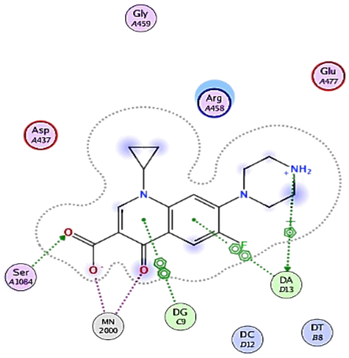

X-Ray Crystal Structure Determinations of 3a and 3b

The single-crystal X-ray diffraction study were carried out on a Rigaku XtaLAB Synergy R diffractometer with HyPix-Arc 100 detector at 120(2) K (using Cu-K□ radiation (□ = 1.54178 Å, PhotonJet R rotating anode generator). Dual space methods (SHELXT) [G. M. Sheldrick, *Acta Crystallogr.* 2015, **A71**, 3-8] were used for structure solution and refinement was carried out using SHELXL-2014 (full-matrix least-squares on F^2) [G. M. Sheldrick, *Acta Crystallogr.* 2015, **C71**, 3-8]. Hydrogen atoms were localized by difference electron density determination and refined using a riding model (H(N) free). Semi-empirical absorption corrections were applied. In **3b** one furan-2-yl moiety was disordered (see cif-file for detaila). **3b** was refined as a 2-component twin (see cif-file for details).

3a - SB1526_HY: orange crystals, $C_{19}H_{16}N_2O_5$, M_r = 352.34, crystal size 0.10 × 0.08 × 0.04 mm, triclinic, space group *P-1* (No. 2), a = 8.2266(2) Å, b = 8.5847(3) Å, c = 12.5866(3) Å, α = 106.141(3)°, β = 103.673(2)°, γ = 100.320(2)°, V = 800.60(4) Å^3, Z = 2, ρ = 1.462 Mg/m^{-3}, μ(Cu-K$_\alpha$) = 0.90 mm^{-1}, F(000) = 368, T = 120(2) K, $2\theta_{max}$ = 158.6°, 17010 reflections, of which 3430 were independent (R_{int} = 0.024), 244 parameters, 3 restraints, R_1 = 0.039 (for 3087 I > 2σ(I)), wR_2 = 0.116 (all data), S = 1.07, largest diff. peak / hole = 0.34 / -0.26 e Å^{-3}.
3b – SB1525_HY: orange crystals, $C_{20}H_{18}N_2O_5$, M_r = 366.36, crystal size 0.14 × 0.10 × 0.03 mm, triclinic, space group *P-1* (No. 2), a = 7.9731(2) Å, b = 8.1795(2) Å, c = 28.7599(7) Å, α = 91.662(2)°, β = 90.972(2)°, γ = 115.979(2)°, V = 1684.45(8) Å^3, Z = 4, ρ = 1.445 Mg/m^{-3}, μ(Cu-K$_\alpha$) = 0.87 mm^{-1}, F(000) = 768, T = 120(2) K, $2\theta_{max}$ = 158.4°, 9888 independent reflection (R_{int} = 0.000, using a HKLF 5 file due to twin refinement), 495 parameters, 34 restraints, R_1 = 0.051 (for 8552 I > 2σ(I)), wR_2 = 0.142 (all data), S = 1.06, largest diff. peak / hole = 0.30 / -0.30 e Å^{-3}
CCDC 2312660 (**3a – SB1526_HY**) and 2312661 (**3b – SB1525_HY**) contain the supplementary crystallographic data for this paper. These data can be obtained free of charge from The Cambridge Crystallographic Data Centre via www.ccdc.cam.ac.uk/data_request/cif.

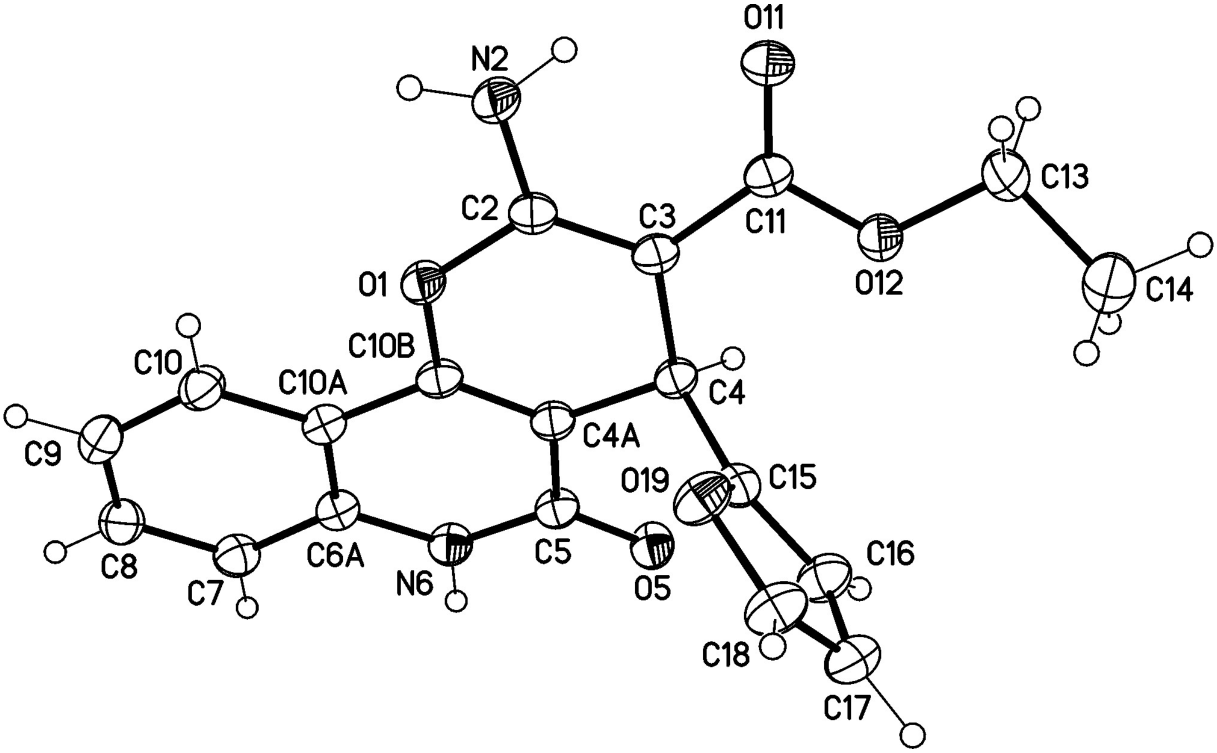

Fig. 31x. Molecular structure of **3a – SB1526_HY** (displacement parameters are drawn at 50 % probability level).

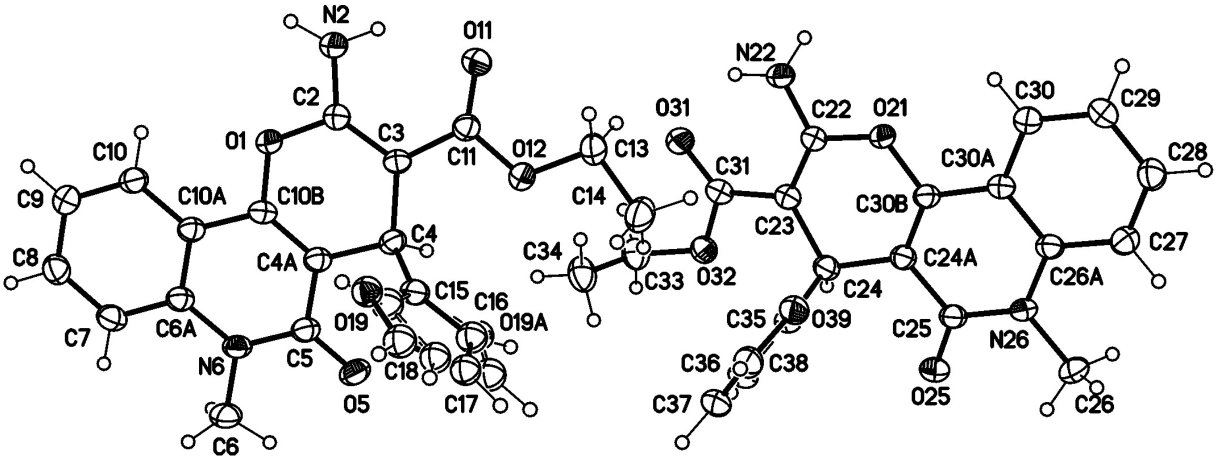

Fig. 32x. Molecular structure (asymmetric unit) of **3b – SB1525_HY** (displacement parameters are drawn at 50 % probability level).

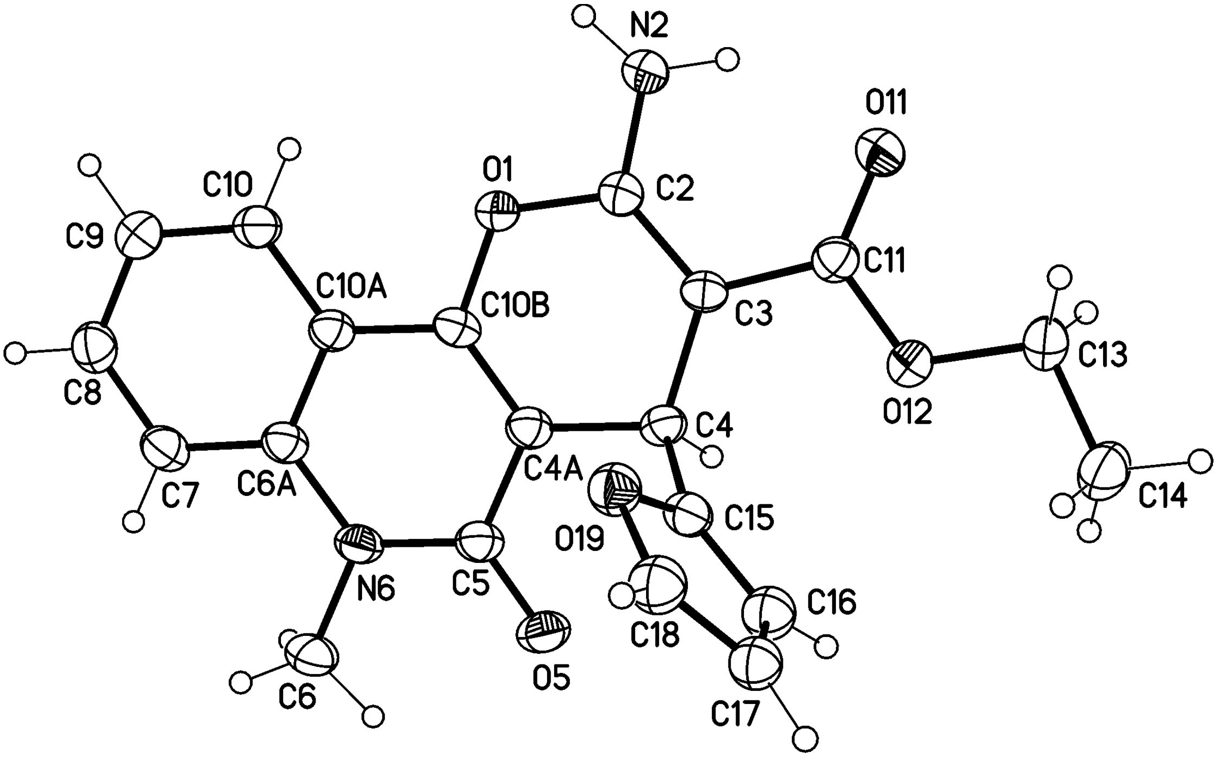

Fig. 33x. 1st crystallographic independent molecule of **3b – SB1525_HY** (displacement parameters are drawn at 50 % probability level).

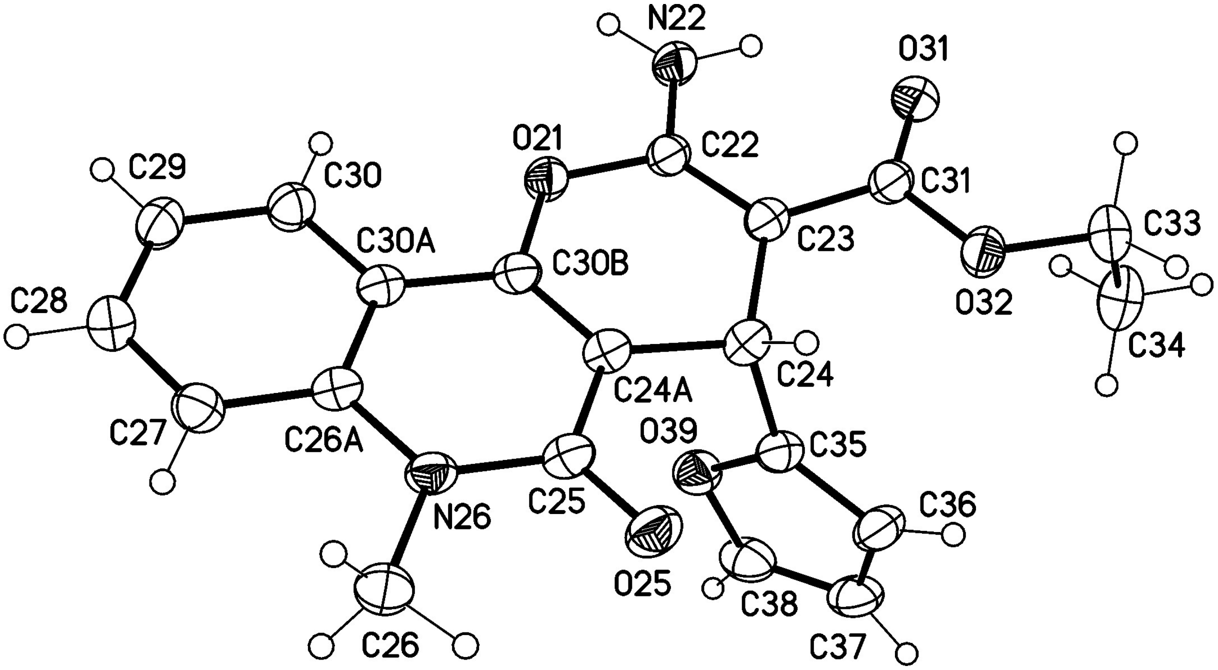

Fig. 34x. 2nd crystallographic independent molecule of **3b – SB1525_HY** (displacement parameters are drawn at 50 % probability level).
